# Vesicular Carriers for Phytochemical Delivery: A Comprehensive Review of Techniques and Applications

**DOI:** 10.3390/pharmaceutics17040464

**Published:** 2025-04-02

**Authors:** Shery Jacob, Fathima Sheik Kather, Sai H. S. Boddu, Rekha Rao, Anroop B. Nair

**Affiliations:** 1 Department of Pharmaceutical Sciences, College of Pharmacy, Gulf Medical University, Ajman 4184, United Arab Emirates; fathima.sheik@gmu.ac.ae; 2 Department of Pharmaceutical Sciences, College of Pharmacy and Health Sciences, Ajman University, Ajman 346, United Arab Emirates; s.boddu@ajman.ac.ae; 3 Center of Medical and Bio-Allied Health Sciences Research, Ajman University, Ajman 346, United Arab Emirates; 4 Department of Pharmaceutical Sciences, Guru Jambheshwar University of Science and Technology, Hisar 125001, India; rekhaline@gjust.org; 5 Department of Pharmaceutical Sciences, College of Clinical Pharmacy, King Faisal University, Al-Ahsa 31982, Saudi Arabia; anair@kfu.edu.sa

**Keywords:** phytochemical, natural extracts, nanovesicles, phytosomes, delivery systems, preclinical evaluation, clinical trials, patents

## Abstract

Natural substances, especially those derived from plants, exhibit a diverse range of therapeutic benefits, such as antioxidant, anti-inflammatory, anticancer, and antimicrobial effects. Nevertheless, their use in clinical settings is frequently impeded by inadequate solubility, limited bioavailability, and instability. Nanovesicular carriers, such as liposomes, niosomes, ethosomes, transferosomes, transethosomes, and cubosomes, have emerged as innovative phytochemical delivery systems to address these limitations. This review highlights recent developments in vesicular nanocarriers for phytochemical delivery, emphasizing preparation techniques, composition, therapeutic applications, and the future potential of these systems. Phytosomes, along with their key advantages and various preparation techniques, are extensively described. Various in vitro and in vivo characterization techniques utilized for evaluating these nanovesicular carriers are summarized. Completed clinical trials and patents granted for nanovesicles encapsulating phytochemicals designed for systemic delivery are tabulated. Phytochemical delivery via vesicular carriers faces challenges such as low stability, limited active loading, scalability issues, and high production costs. Additionally, immune clearance and regulatory hurdles hinder clinical application, requiring improved carrier design and formulation techniques.

## 1. Introduction

Natural products, particularly those derived from plants, owing to their diverse and significant biological activities have long been regarded as key resources for treating various diseases. Phytoconstituents are bioactive compounds naturally found in plants, responsible for their therapeutic properties and protective roles, and have been utilized in both traditional and modern medicine. Produced as secondary metabolites, they help plants resist environmental stressors, pathogens, and herbivores [1]. Phytochemicals, in their native form or as metabolites, provide health benefits by scavenging free radicals, chelating metals, inhibiting microtubule and microfilament assembly, and blocking protease activity [2].

Polyphenols, flavonoids, carotenoids, and allyl sulfide-containing compounds are renowned for their antioxidant and anti-inflammatory effects, aiding in the prevention and management of chronic diseases [3,4]. Isoflavones, particularly those derived from soy, function as phytoestrogens, mimicking hormonal activity and offering benefits in conditions like menopause [5]. Protease inhibitors and indole-containing compounds play crucial roles in enzyme regulation, contributing to cellular homeostasis and defense mechanisms. Proanthocyanidins serve as potent anti-infective agents, while saponins and capsaicin have been identified as modulators of DNA replication, indicating potential in cancer therapy [6]. Additionally, curcuminoids such as curcumin exhibit a wide spectrum of activities, including antitumor, antioxidant, and anti-inflammatory properties, making them valuable in the treatment of various ailments [7,8]. Natural compounds like piperine, caraway, curcumin, and genistein act as bioenhancers by increasing the bioavailability of drugs when taken orally [9]. Bioenhancers are agents that boost the therapeutic efficacy of a drug by increasing its concentration in biological fluids or systemic circulation, often by enhancing the absorption or bioavailability of the active compound [10]. Reducing therapeutic drug dosage can lower treatment costs, minimize side effects, and help prevent drug resistance in long-term diseases like cancer and infections [11]. Bioenhancers, such as piperine, can possess therapeutic properties, demonstrating effectiveness in treating diseases like diabetes, arthritis, cancer, and cardiovascular conditions [12]. Herbal bioenhancers hold promise in combating multifactorial diseases like cancer by overcoming resistance mechanisms and boosting the effectiveness of anticancer drugs [9]. In antimicrobial therapy, they offer hope against antibiotic resistance by enhancing the efficacy of antimicrobial agents [9]. This is achieved through mechanisms such as obstructing efflux pumps, preventing the formation of biofilm, modulating virulence factors, and modifying host defense mechanisms. The primary action of herbal bioenhancers is their ability to inhibit P-glycoprotein and metabolic enzymes such as cytochrome P450 enzymes and Uridine 5′-diphosphate-glucuronosyltransferase [13,14]. Thus, bioenhancers function by retaining the drug molecule within the cell without altering its metabolic activity. It was reported that piperine significantly enhances the bioavailability of the anticancer drug docetaxel by inhibiting the metabolizing enzyme CYP3A4, which led to an increase in the drug’s C_max_ from 6808 ng/mL to 11,380 ng/mL [15]. Similarly, it was found that the bioavailability of tamoxifen was improved in rats pretreated with either curcumin or piperine [16]. Increased drug absorption, bypassing of hepatic first-pass metabolism, enhanced drug retention, and reduced multidrug resistance are the resulting effects of combining anticancer drugs with bioenhancers in the treatment of various cancers, leading to improved bioavailability. Moreover, various medicinal plants have been used for treating skin disorders [17].

Phytochemical delivery faces several significant challenges that limit their clinical translation and therapeutic potential. In addition to being broken down by enzymes in the gastrointestinal tract (GIT), the polarity of these molecules along with their size make it difficult for them to cross the blood–brain barrier (BBB), the inner endothelial lining of blood vessels, and the GIT and mucosa [18]. Furthermore, their clinical application is often limited by challenges such as low aqueous solubility, fast metabolism, low bioavailability, and elimination, instability and short duration of action [19]. This necessitates administering a higher dose of phytochemicals, which can potentially increase peripheral organ toxicity. Variable pharmacokinetics among individuals and extensive first-pass metabolism further contribute to inconsistent therapeutic outcomes. Moreover, conventional formulations often lack targeted delivery capabilities, reducing their effectiveness at the desired site of action. Collectively, these challenges underscore the need for advanced drug delivery strategies to enhance the clinical utility of phytochemicals.

The diverse therapeutic potential of phytochemicals has spurred significant interest in pharmaceutical technology, leading to the development of formulations that enhance their bioavailability and efficacy, thereby maximizing their health benefits [20]. To address the limitations associated with phytochemical delivery, a range of advanced drug delivery systems have been explored. The incorporation of these natural products into polymeric and lipid nanocarriers has emerged as a novel strategy to overcome these hurdles, offering enhanced delivery and efficacy [21]. Nanocarriers have several advantages, including protection from environmental degradation, enhanced solubility, and improved bioavailability. Incorporating stabilizers such as cholesterol and employing lyophilization technique have been shown to enhance the stability of advanced vesicular formulations, including transethosomes [22]. By providing an increased surface area, nanocarriers facilitate better absorption and penetration through biological barriers. Additionally, they provide sustained as well as controlled drug release, thus maintaining therapeutic levels over an extended time while minimizing systemic side effects through site-specific delivery [23]. Functionalized nanocarriers have further advanced the field by incorporating surface modifications and ligand conjugations can further improve site-specific delivery and cellular uptake [24]. Surface modification approaches, including PEGylation, have been utilized to enhance the stability and prolong the circulation time of nanosystems. By forming a steric barrier around nanoparticles, PEGylation minimizes protein adsorption and reduces recognition by macrophages, thus improving their pharmacokinetic profile [25]. These innovations enhance stability, entrapment efficiency, and therapeutic efficacy while addressing toxicity concerns [26]. Stimuli-responsive nanocarriers represent the next frontier, offering precise, adaptive drug delivery tailored to the needs of specific therapeutic applications [27]. Lipid–polymer hybrid nanoparticles are promising drug delivery systems that combine the biocompatibility of lipid nanosystems with the structural stability of polymers. They enable the co-delivery of amphiphilic molecules and have been effectively used to transport both phytochemicals and chemotherapeutic agents for enhanced cytotoxicity against cancer. By targeting tumor sites and enabling controlled drug release, hybrid nanocarriers improve therapeutic efficacy and chemo-sensitization of cancer cells [28]. Targeting ligands such as folic acid enhance cellular uptake through receptor-mediated endocytosis. Folic acid, which selectively binds to folate receptors, which are overexpressed in many tumors, helps reduce off-target effects on normal tissues. Ligand-functionalized pH-sensitive NPs were developed for co-delivery of carboplatin and paclitaxel in cervical cancer treatment [29]. These nanoparticles exhibited a uniform size (~170 nm), high cellular uptake (66.7%), and strong cell inhibition (23%). They also demonstrated pH-responsive drug release and a synergistic therapeutic effect of both drugs against cervical cancer cells.

Encapsulation of phytochemicals also helps bypass first-pass metabolism and improve systemic circulation time. Nanocarriers enhance the ability of natural products to cross the blood–brain barrier and facilitate their uptake into cancer cells via endocytosis, improving therapeutic potential. Scalable production of vesicular nanocarriers has advanced through technologies like microfluidics, high-pressure homogenization, continuous manufacturing, and bioreactor systems. These methods enhance control, uniformity, and practical yield, addressing limitations in large-scale production and supporting the clinical translation of nanocarrier-based drug delivery systems [30,31,32]. This integration of natural products into nanocarriers not only addresses their intrinsic limitations but also represents an important leap in drug delivery technology, paving the way for safer, more effective, and targeted treatments.

The aim of this review was to highlight both phyto-phospholipid complexes and advanced vesicular delivery systems such as liposomes, invasomes, niosomes, bilosomes, transfersomes, ethosomes, transethosomes, and cubosomes that have been extensively studied for enhancing the bioavailability, stability, and therapeutic effectiveness of phytochemicals. Vesicular carriers with limited evidence of application in phytochemical delivery or lacking relevance in current research trends were excluded to maintain focus and coherence. Although no strict exclusion criteria were applied, preference was given to systems supported by peer-reviewed publications, preclinical studies, clinical trials, or patent literature.

## 2. Bioavailability and Stability Challenges

Phytochemicals exhibit diverse physicochemical properties, which contribute to alteration in their solubility, bioavailability, and chemical stability profiles. The details of the most frequently investigated phytochemicals are summarized in Table 1.

### 2.1. Bioavailability

Applying the Lipinski rule of five to phytochemicals can help determine the drug likeness of these natural compounds for product development, similar to active pharmaceutical ingredients. Many natural compounds, such as phytochemicals, may have larger molecular weights, which limit their solubility and permeability [33]. When they exceed this threshold, modifications or nanoformulations might be needed to enhance their delivery. Excessive hydrogen bond donors in natural compounds, such as hydroxyl or amine groups, can hinder permeability across biological membranes. Similarly, a high number of acceptors, such as oxygen or nitrogen atoms, can restrict passive diffusion, necessitating careful structural modifications or encapsulation strategies. A compound’s lipophilicity (LogP) determines its solubility and permeability. For instance, lipophilic compounds like terpenes often face challenges with water solubility, limiting their gut absorption. Strategies such as incorporating lipid nanosystems, including solid lipid nanoparticles (SLNs) or self-emulsifying systems, are commonly employed to enhance their bioavailability [34,35]. Moreover, decreased aqueous solubility often necessitates the use of solvent-based extraction methods for isolating natural compounds from medicinal plants. Oral delivery of bioactive phytochemicals faces challenges such as the viscosity of GI contents and dietary intake, leading to the exploration of alternative delivery forms to enhance bioavailability [36]. In the GIT, phytochemicals undergo enzymatic metabolism in different regions, which can either reduce their activity or improve bioavailability compared to the parent compound [37,38].

Bioefficacy of phytochemicals refers to the ability of these compounds to exert beneficial biological effects in the body after they have been absorbed and metabolized, which depends on their bioaccessibility and bioavailability [38]. It is assessed through short-term changes in biomarkers like plasma lipid levels, glucose, blood pressure, antioxidant activity, and liver function. Bioaccessibility of phytochemicals refers to the extent and rate at which the active ingredients in phytochemicals become available for absorption in the GIT after ingestion [37]. This is dependent on various factors such as the chemical nature, polarity, and solubility of the phytochemicals, and how they interact with food components. After absorption, small intestinal cells enable cellular uptake and efflux, while hepatic cells transform inactive precursors into active forms, crucial for bioaccessibility [18]. Additionally, the microbiota in the large intestine metabolizes unabsorbed phytochemicals, impacting bioefficacy.

### 2.2. Stability in Solid State

Phytochemicals in solid state and aqueous solutions are influenced by environmental, chemical, and physical factors that alter the stability of the compounds. The presence of moisture/humidity can accelerate hydrolysis, particularly in compounds with ester and amide linkages. Elevated temperatures can increase the rate of chemical reactions, leading to faster degradation due to increased collision frequency, as described by the Arrhenius equation. L-ascorbic acid stability is affected by oxygen, light, and temperature [39]. Phytochemicals, namely procyanidin B2, (-)epicatechin, chlorogenic acid, hyperoside, and isoquercetin, remained stable for six months at 4 °C but degraded rapidly at 40 °C [40].

The stability of phenolic compounds following extraction from plant matrices is critical, as they are prone to degradation when exposed to the external environment (oxygen or thermal stress) [41]. The conjugated system of double bonds in carotenoids is susceptible to isomerization and oxidation, particularly during thermal processing due to high temperatures and oxygen exposure [42]. UV or visible light can induce photochemical degradation, and the presence of oxygen can promote oxidative degradation, particularly in compounds sensitive to oxidation [43]. Surface pH changes (even in the solid state) can influence the stability of sensitive molecules [44]. Different polymorphic forms of drugs may exhibit varying stability profiles, with some forms being more prone to degradation [45]. Smaller particle sizes increase the surface area exposed to environmental factors, enhancing the degradation rate. Excipients in formulations may interact with phytochemicals, leading to instability through chemical or physical interactions [46]. Residual solvents, catalysts, or trace metals can trigger degradation pathways such as hydrolysis or oxidation [47].

### 2.3. Stability in Aqueous Solution

Factors influencing degradation in aqueous solutions include pH, where acidic or basic conditions can accelerate hydrolysis, oxidation, or ionization. The stability of phenolic compounds is strongly influenced by pH and their structural conformation. Flavan-3-ols are highly stable in acidic conditions but become unstable at a neutral pH [48]. Higher temperatures increase molecular mobility, enhancing degradation. UV or visible light can cause photochemical degradation, and dissolved oxygen promotes photooxidation, especially in unsaturated or phenolic compounds. It was reported that thymoquinone, a potent anticancer phytochemical, is highly soluble in aqueous solutions (500 µg/mL) but remains unstable, with its stability significantly influenced by pH and light, the latter being more critical [49]. This instability makes pure aqueous solutions unsuitable as pharmaceutical carriers for drugs, suggesting that a combination of organic and aqueous solvents is a more effective alternative [49]. High ionic strength affects solubility and stability, while the solvent composition influences reactivity and overall stability [50]. Complexation with metal ions can lead to degradation, and residual catalysts or impurities can accelerate processes like oxidation or hydrolysis [51]. Additionally, higher concentrations of phytochemicals may promote aggregation or precipitation, impacting stability [52].

## 3. Functional Role of Nanoparticles in Phytochemical Delivery Systems

Nanoparticles are highly effective drug delivery carriers, owing to their submicron particle size and distinctive physicochemical properties that facilitate targeted tissue delivery [53,54]. These versatile carriers are available as particulate, soluble, or target-specific recognition moieties, which can encapsulate a wide variety of drugs. They are fabricated using diverse polymers and lipids utilizing a range of manufacturing techniques [18]. The composition of the carrier and the entrapping agent plays an important role in determining permeability, release rate, mucoadhesion, and targeting efficiency. Nanocarriers are typically formulated as aqueous dispersions or incorporated into gel or film matrices.

**Figure 1:**
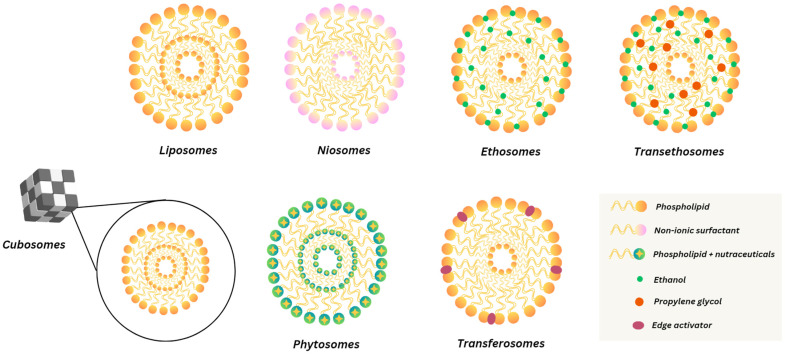
Different types of nanovesicular carriers utilized for the delivery of phytochemicals.

Vesicular nanocarriers with organized bilayer structures enable the effective encapsulation and delivery of hydrophilic and lipophilic drugs. These vesicular systems are used to encapsulate and deliver various phytoconstituents with enhanced efficiency [55,56,57]. They offer several advantages, including controlled release, targeted delivery, improved stability, and biocompatibility, making them suitable for various administration routes. A comparison of various nanovesicular carriers, summarizing their composition, advantages, limitations, key attributes, and stability are presented in Table 2 and illustrated in Figure 1. However, these are subject to challenges such as limited drug-loading capacity, high production costs, stability issues, and scalability constraints persist. Recent advances, including stimuli-responsive vesicles, and polymer–lipid hybrid systems have significantly improved their functionality and therapeutic potential [58,59]. These innovations continue to position vesicular systems as an efficient nanocarrier in modern pharmaceutical research for achieving better patient outcomes.

### 3.1. Phytosomes

Phytosome^®^ or phyto-phospholipid complexes are well-defined systems formed by the association of phytochemicals (usually polyphenols) with phospholipids, typically phosphatidylcholine, in a defined stoichiometric ratio. This complex results in improved solubility, stability, skin/mucosal permeation, and bioavailability of the active compound. Nanophytosomes are phyto-phospholipid complexes under 200 nm in size, designed to enhance absorption and biological activity, with composition similar to regular phytosomes but improved delivery due to their nanoscale size. The formation of phytosomes involves interaction between the hydrophilic phytoconstituent and the phospholipid through hydrogen bonding and van der Waals forces. Among all phytochemicals, only those containing an active hydrogen atom (e.g., -COOH, -OH, -NH_2_, -NH), such as polyphenols, can be incorporated into a phytosome structure [60]. This active hydrogen atom enables hydrogen bond formation between the phytochemicals and the hydrophilic groups of the amphiphilic phospholipids [61]. It was found that hydroxyl groups present in polyphenols can efficiently interact with the nitrate and phosphate groups present in phospholipids. The binding results in the formation of a lipid-compatible molecular complex, where the hydrophilic head of the phospholipid binds to the phytoconstituent, and the lipophilic tail interacts with cell membranes [62]. Structurally, phytosomes resemble biological cell membranes due to the presence of phospholipids, which facilitate the absorption of active compounds [63,64]. Phytosomes are amphiphilic with the ability to encapsulate hydrophilic and lipophilic phytochemicals, providing stability and protection. Phytosomes are preferred for transporting poorly water-soluble flavonoids, such as curcumin, polyphenol, and quercetin, that often struggle to cross lipid-rich biological membranes [65,66]. Phospholipid complexes of curcumin achieve higher bioavailability of the parent compound compared to uncomplexed curcumin [66]. Indena^®^ has developed a wide range of phytosome-based formulations aimed at enhancing the bioavailability and therapeutic efficacy of various phytochemicals. Notable products include Siliphos^®^ (from *Silybum marianum*) [67] for hepatoprotective and antioxidant effects, Hawthorn phytosome (*Crataegus* flavonoids) with cardioprotective and antihypertensive properties, and Oleaselect™ Phytosome (from *Olea europaea*) for its anti-inflammatory and antihyperlipidemic benefits. Polinacea™ phytosome (*Echinacea angustifolia*) [68] and Ginseng phytosome (*Panax ginseng*) support immunomodulation [69], while Ubiqsome^®^ phytosome delivers CoQ10 for mitochondrial and antioxidant activity [70]. Quercefit™ [71] and Greenselect^®^ [72] target antioxidant and metabolic health, whereas Vazguard™ (bergamot extract) aids in regulating cholesterol and glucose levels. Other formulations, such as Casperome^®^ (*Boswellia serrata*) [73], curcumin phytosome [74], Leucoselect^®^ (grape seed) [75], and Virtiva^®^ (*Ginkgo biloba*) [76], contribute to anti-inflammatory, joint, cardiovascular, and cognitive health. Additional products like Centevita^®^ (*Centella asiatica*) [77], Soyselect^®^ (soybean extract) [78], and Visnadex (*Amni visnaga*) [79] further extend applications to skin health, hormone balance, and microcirculation. These innovations reflect Indena’s role in developing clinically relevant, plant-based vesicular delivery systems.

A variety of solvents have been used as reaction mediums to formulate phyto-phospholipid complexes. Aprotic solvents, such as methylene chloride and ethyl acetate, do not form hydrogen bonds, while protic solvents like ethanol and methanol contain hydrogen atoms attached to electronegative atoms, making them more effective in yielding complexes with lower residues. Ethanol is commonly used for its high efficiency, while some studies also utilize mixed solvent systems, such as dichloromethane and methanol or water and diethyl ether, to dissolve phospholipids separately from the drug or extract [80,81].

### 3.2. Liposomes

Liposomes are spherical, bilayered vesicles, composed of phospholipids and cholesterol, capable of delivering hydrophilic drugs (within their aqueous core) and hydrophobic drugs (within their lipid bilayer). Due to their structural versatility and biocompatibility, liposomes have become a prominent carrier system in drug delivery [82]. They offer advantages such as biocompatibility, targeted delivery, improved drug stability, and reduced systemic toxicity, but face challenges like short circulation time, high production costs, and stability issues. Recent advances include PEGylated “stealth” liposomes, stimuli responsive designs, liposomal vaccines, and functionalized liposomes for active targeting. Liposomes are commonly categorized based on their structural characteristics, composition, and method of preparation [83]. Liposomes are administered via oral, parenteral, pulmonary and ocular routes, with each offering distinct advantages for drug delivery.

Liposomes play a pivotal role in the delivery of phytochemicals, which are often limited by low bioavailability, poor water solubility, and rapid metabolism [84]. By encapsulating phytoconstituents, such as curcumin, quercetin, betulinic acid, artemisinin, or resveratrol, liposomes enhance their stability, solubility, and targeted delivery to the desired site of action. They also improve the therapeutic efficacy of these compounds by ensuring sustained release and protection from degradation. Liposomes formulated with dioleoylphosphatidylcholine (DOPC) significantly improve curcumin’s solubility and stability. However, curcumin is quickly released in the presence of serum or cells, limiting the formulation’s effectiveness in vivo [85]. Quercetin, a potent natural antioxidant, was encapsulated in Eudragit-coated liposomes designed for targeted intestinal delivery. The Eudragit coating protected the liposomes from gastric conditions and enhanced their stability in simulated gastrointestinal fluids. Importantly, quercetin’s antioxidant activity remained intact, effectively inhibiting DPPH radicals and reducing reactive oxygen species (ROS) levels in human intestinal cells [86]. PEGylated liposomes enhance drug delivery by prolonging circulation time, reducing immune clearance, and improving drug accumulation at target sites, especially tumors. This results in increased stability, bioavailability, and therapeutic efficacy. PEGylated liposomes loaded with betulinic acid (BA) were developed to enhance drug delivery performance. These liposomes demonstrated a more sustained drug release over 72 h (89%) compared to conventional BA liposomes (78%). In vivo studies showed significantly greater tumor growth inhibition (64.3%) with the PEGylated formulation, highlighting its improved delivery efficiency, prolonged release, and enhanced anticancer activity over free BA or standard liposomes [87]. A liposome-based formulation containing soy phosphatidylcholine and cholesterol in a 3:1 ratio, combined with a biosurfactant (10% MEL-A) and 1% betulinic acid (BA), significantly inhibited HepG-2 cell proliferation, demonstrating strong anticancer activity. Additionally, it improved the solubility, stability, and bioavailability of the active compounds and has already been introduced into clinical use [88]. Dihydroartemisinin was encapsulated in both conventional and long-circulating liposomes and evaluated for cytotoxicity against MCF-7 breast cancer cells. Both formulations showed suitable size for parenteral use and achieved high encapsulation efficiency (~70%). They remained physically and chemically stable during storage and in the presence of albumin. Flow cytometry revealed efficient cellular uptake, with conventional liposomes showing greater internalization than long-circulating ones. Cytotoxicity studies showed IC_50_ values of 12.1 μM for free dihydroartemisinin, and 48.2 μM and 77.0 μM for the conventional and long-circulating liposomes, respectively [89]. Liposomes serve as an effective delivery system for resveratrol, as they help maintain its active trans-form by preventing conversion to the less effective cis-form. Resveratrol tends to localize at the liposome surface, allowing for controlled, sustained release that avoids cellular membrane overload and reduces cytotoxicity. This enables the use of drug concentrations that would otherwise be toxic in free form. Additionally, the liposomal bilayers enhance phytochemicals’ long-term stability and support the activation of cellular defense mechanisms [90,91]. Recent studies have highlighted liposomal formulations as effective systems for enhancing the bioactivity of herbal extracts and phytochemicals in treating chronic diseases, including cancer, inflammation, eye and skin disease, malaria, osteosarcomas and neurodegenerative disorders [92,93,94].

Liposomes are among the most effective lipid-based entrapment systems due to their ability to use natural materials, encapsulate phytochemicals with varying solubilities, and protect ingredients from oxidation by metal ions, free radicals, and enzymes [95]. They are well known for enhancing the cutaneous delivery of molecules of various sizes and have been recently utilized for skin delivery of phytochemicals due to their high loading efficiency, carrier capacity, and compatibility with different skin layers. The term ‘phospholipid vesicles’ is a broader term encompassing any vesicular system composed of phospholipids, such as liposomes, transferosomes, ethosomes, cubosomes, and phytosomes. Not all phospholipid vesicles are phytosomes, but all phytosomes fall under this general category. Phyto-phospholipid complex formulations are extensively studied for addressing bioavailability challenges by forming molecular complexes with phytochemicals, rather than encapsulating them in phospholipid vesicles [96].

The potential of phospholipid vesicles as carriers for topical and transdermal delivery of phytochemicals is described in a recent review [97]. Modified liposomes, such as glycerosomes, utilize glycerol as a partial penetration enhancer to improve the skin bioavailability of encapsulated phytochemicals, including extracts and oils, compared to traditional liposomes [98]. The moisturizing effect of glycerol disrupts the ordered structure of the stratum corneum, facilitating the accumulation of bioactive phytochemicals in various skin layers. Glycerosomes modified with maltodextrin and polymers, like gelatin forming gluglycerosomes, demonstrated improved stability, viscosity, and effectiveness in delivering *Hypericum scruglii* extract to wound sites [99]. Additionally, glycerosomes have been utilized to encapsulate *Thymus capitatus* oil for treating oral cavity diseases [100] and *Rosmarinus officinalis* extract to enhance the stability of antioxidant polyphenols [95]. Modifications with sodium hyaluronate to create gly-hyalurosomes, loaded with *Citrus limon* var. *pompia* extract, effectively prevented oxidative damage to keratinocytes and fibroblasts while supporting their viability [101]. Table 3 depicts liposomes encapsulating selected phytochemicals, including their preparation techniques, composition, and key features.

### 3.3. Invasomes

Invasomes are modified liposomes composed of phospholipids, ethanol, terpenes, and water, and may also include lysophosphatidylcholine. Frequently, phospholipids such as phosphatidylcholine and egg or soy lecithin form the bilayer of the vesicle; lysophosphatidylcholine acts as an edge activator, enhancing vesicle flexibility; meanwhile, terpenes, such as eugenol, thymol, limonene, citral, cineole, nerolidol, fenchone, anethole, geraniol, and linalool, along with ethanol, serve as penetration enhancers and contribute to the fluidity of the vesicle bilayer [127]. Other phospholipids, such as phosphatidylserine, phosphatidic acid, phosphatidylglycerol, and phosphatidylinositol, are less commonly used and typically depend on specific formulation requirements. Since these phospholipids are similar to those found in mammalian cells, they can adhere to the cell surface either nonspecifically or through specific interactions with cell surface receptors. The internalization of liposomes by target cells typically occurs via endocytosis. Other mechanisms include cell vesicle fusion and lipid exchange cell surface receptors. Ethanol and terpenes enhance invasome performance for transdermal drug delivery by disrupting the tightly packed lipid structure of the stratum corneum. Ethanol fluidizes the lipid bilayer, increases vesicle flexibility, and prevents aggregation by providing a negative surface charge. Terpenes act as penetration enhancers by breaking lipid packing in the outermost layer, improving drug partitioning, and opening polar pathways for deeper penetration. Both components facilitate vesicle deformation during skin permeation without rupture or drug loss. The effectiveness of terpenes depends on properties like molecular size, lipophilicity, and degree of unsaturation, and they are recognized as safe by the US FDA [127,128]. Membrane flexibility can promote vesicle aggregation and the formation of multilamellar onion-like structures. However, flexible vesicles are more sensitive to temperature changes, leading to reduced stability during storage. Terpenes such as limonene increased membrane rigidity of the vesicles, possibly due to competitive interactions with cholesterol.

Polar phospholipid-based invasomes, with their adaptability to low water content conditions and ultra flexibility, penetrate deeper skin layers by leveraging the transepidermal osmotic gradient and elastomechanics, similar to transferosomes. While effective for dermal and transdermal drug delivery, formulation challenges like oxidative degradation, hydrolysis, and leakage of encapsulated bioactives must be addressed [128]. To improve patient compliance and prolong drug retention, these carriers are often converted into invasome gels using gelling agents such as carbomers (Carbopol 934, 940), poloxamer 188, and HPMC K4M. Capsaicin is primarily found in plants of the *Capsicum* genus, which belongs to the *Solanaceae* family. Capsaicin is widely used in the pharmaceutical field for pain management, including neuropathic and musculoskeletal pain, due to its ability to desensitize TRPV1 receptors. It also exhibits anti-inflammatory, antimicrobial, and anticancer properties, with potential applications in weight management and respiratory conditions. Additionally, capsaicin is used in dermatology for relieving itchiness and inflammation in conditions like psoriasis. An investigation was carried out to prepare capsaicin-loaded (0.15% *w*/*w*) invasomes, which exhibited a size < 100 nm, a narrow size distribution (PDI ~0.01–0.30), and a slightly negative zeta potential < −20 mV [129]. Capsaicin-loaded invasomes demonstrated enhanced skin permeability compared to conventional liposomes and a commercial ethanolic solution (0.15%).

A study developed curcumin-loaded terpenoid invasomal nanovesicles using a mechanical dispersion process with eucalyptol as a permeation enhancer [130]. Transmission electron microscopy revealed spherical vesicles with a size of 461.57 ± 1.38 nm and an EE of 80.54 ± 0.38%. The in vitro release followed the Higuchi kinetic model, and ex vivo studies showed that the optimized formulation achieved two and a half times higher curcumin permeation across pig ear skin compared to a curcumin solution, with a flux of 179.44 ± 0.26 µg/cm^2^/h. Berberine-loaded invasomes formulated with 2% Carbopol gel exhibited superior analgesic and antiarthritic activity compared to the standard gel (Omnigel, 0.1%), providing prolonged effects [131]. Radiographic analysis of hind paw rats in the complete Freund’s adjuvant model revealed bone resorption, reduced joint gaps, and significant connective tissue expansion following treatment with berberine-loaded invasomal gel. Histology of ankle joints further confirmed the gel’s enhanced penetration capability and its profound therapeutic effects. Curcumin-loaded invasomes were developed using a factorial design to assess the impact of terpene type and concentration on formulation properties [132]. The optimized invasomal formulation, with an EE of 85.84 ± 0.56% and vesicle size of 302.33 ± 1.53 nm, was incorporated into a topical gel. This invasomal gel exhibited three times higher permeation flux than a plain gel. In vivo studies in psoriatic BALB/c mice showed that the invasomal gel significantly accelerated and improved recovery compared to conventional curcumin gel. In another attempt, the delivery of dapsone using invasomes for acne therapy was investigated in rats. The results here signify greater skin retention by the developed carrier [133]. Similarly, Castangia et al. developed santosomes, vesicles formulated with *Santolina insularis* essential oil, propylene glycol and phosphatidylcholine. Santosomes, loaded with the wound healing protein phycocyanin, demonstrated enhanced drug delivery and wound healing potential due to the synergistic action of terpenes and phospholipids in the essential oil [134].

### 3.4. Niosomes

Niosomes are self-assembled vesicles of nonionic surfactants in an aqueous phase, mostly in the presence of cholesterol or other stabilizing agents. These vesicles are structurally similar to liposomes but are more stable and cost-effective, making them a potential drug delivery system. Niosomes can encapsulate both hydrophilic and hydrophobic drugs, enhancing their stability, bioavailability, and therapeutic efficacy [135,136,137]. Niosomes are osmotically responsive, biocompatible, biodegradable, nontoxic, and possess immune-suppressive properties [138]. Niosomes are widely used in various drug delivery applications, such as cancer therapy, and gene and vaccine delivery. They have been explored as versatile drug carriers suitable for various administration routes, including parenteral, oral, dermal, ophthalmic, transdermal, and pulmonary applications.

Niosomes offer a promising delivery system for phytochemicals with low solubility in bodily fluids. Different types of niosomes have been developed to address the limitations of phytoconstituents effectively [139]. A niosome is known as a drug depot because it has the potential to transfer the drug in a controlled or sustained manner to the targeted site. The potential of this vesicle in oral delivery of *Fumaria officinalis* for antineuropathic and anti-inflammatory effects has been evaluated [140]. The bioguided fractionation, as well as phytochemical analysis, identified the alkaloid rich fraction as the major active ingredient. In vivo studies of the optimized formulation (Nio-2) indicate a decrease in TNF-α and Interleukin 6 while enhancing the anti-inflammatory factor IL-10 and ameliorating oxidative stress. The outcome of this investigation confirms the potential of an alkaloid-rich fraction of *Fumaria officinalis* as a new oral therapeutic agent in treating various inflammatory conditions including neuropathic pain. Another study reported the enhancement in solubility and bioavailability of morusin, a flavonoid having anticancer properties when formulated as niosomes [141]. The developed formulation exhibited ideal pharmaceutical characteristics, showed higher cytocompatibility, improved aqueous dispersibility, controlled release, and improved efficacy in four cancer cell lines tested. A study reported in 2020 developed niosomes containing hedera helix extract prepared using common thin film method [142]. The composition has cholesterol (30%) and span 60 (70%) which entrapped the hedera helix extract. The developed formulation exhibits good extract entrapment (~95%), nanosize (~132 nm), and strong negative surface charge with good colloidal stability (−41 mV). The sustained release of extract from niosomes was noticed for 2 days in both cancer and normal conditions. The MTT assay indicates the extract-loaded niosomes were more toxic to the HT-29 cell line.

PEGylated nanosystems offer numerous advantages, making them highly effective in drug delivery. Additionally, PEGylated nanoparticles are adaptable to various administration routes and can integrate multiple functionalities, such as imaging agents and targeting moieties, making them a versatile platform for advanced therapeutic applications. PEGylated niosomes loaded with artemisinin and metformin, prepared using the thin film hydration (TFH) method, were recently investigated [143]. The optimized formulation demonstrated nanosize (256 nm), higher encapsulation efficiency (95%), and appropriate PDI (0.202). PEGylation significantly reduced niosome size due to its hydrophilic properties. In vitro studies revealed dose-dependent toxicity of both free and niosomal formulations, effectively inhibiting the growth of A549 lung cancer cells. RT-PCR results further confirmed that the niosomes exhibited enhanced antiproliferative effects by suppressing antiapoptotic genes and promoting apoptotic gene expression. Table 4 provides an overview of niosomes loaded with selected phytochemicals, detailing their composition, preparation methods, and key characteristics.

### 3.5. Bilosomes

Bilosomes are advanced vesicular carriers made of lipid bilayers stabilized with bile salts, offering enhanced stability and bioavailability for encapsulated compounds. These carriers are designed to withstand the harsh GI environment, including acidic pH and enzymatic degradation, which makes them highly effective for delivering sensitive phytochemicals, such as flavonoids, terpenoids, and polyphenols. The enhanced flexibility of their structure improves transdermal delivery, enabling more effective penetration through the stratum corneum and deeper layers of epidermis and dermis. Moreover, this vesicle enables oral vaccine administration and helps reduce dosing frequency [159]. Due to their potential for controlled and targeted release, they are positioned as valuable tools in the development of functional foods and therapies for chronic diseases [160]. However, they also have limitations, including potential variability in encapsulation efficiency, scalability challenges in manufacturing, and the need for detailed toxicity and long-term safety studies. Despite these challenges, bilosomes remain a promising delivery system for overcoming the inherent limitations of phytochemical bioavailability and stability [161]. It has been reported that bilosomes play a significant role in immunization against infections and diseases by utilizing antigens instead of whole pathogens, enhancing safety [162]. While most bilosome research focuses on oral immunization, recent studies have explored their potential in phytochemical delivery [161]. Notable examples include the use of pharmacologically active polysaccharides from Enteromorpha intestinalis and tripterine from Tripterygium wilfordii for drug delivery. In vivo studies showed longer residence time, improved cellular uptake, and enhanced bioavailability of these compounds [163,164].

Surface coating of bilosomes enhances their stability, functionality, and efficacy in drug delivery [165,166]. It protects the vesicle from degradation in harsh environments, such as the GIT, and ensures prolonged shelf life. Coatings improve mucoadhesion, allowing for increased residence time and better drug absorption, while also providing controlled and sustained drug release. They enhance biocompatibility, reduce immune response, and offer protection against oxidative stress, ensuring the integrity of encapsulated drugs. Furthermore, surface coatings facilitate targeted delivery by enabling functionalization with ligands or antibodies and improve encapsulation efficiency by minimizing drug leakage. These advantages make coated bilosomes particularly effective for oral, ocular, and parenteral drug delivery applications. Chitosan is widely used as a coating agent for nanovesicles, leveraging electrostatic interactions between the negatively charged nanovesicle surface and the positively charged amino groups of chitosan. This coating enhances the stability of nanovesicles, prolongs drug release, increases cellular uptake, and prevents drug leakage [167]. Additionally, chitosan improves mucosal barrier permeability across various tissues, including ocular, intestinal, nasal, and skin barriers [168]. A positive charge on bilosomes facilitates interaction with the negatively charged stratum corneum, making it a promising candidate for delivering berberine to deeper skin layers effectively. Berberine-loaded, chitosan-coated bilosomes were evaluated for their anti-inflammatory efficacy in rheumatoid arthritis management in an animal model [169]. The optimized formulation, containing sodium deoxycholate, had a mean particle size of 202.3 nm with an EE of 83.8%, and zeta potential value of 30.8 mV. It demonstrated a delayed release profile in vitro, enhanced skin permeability ex vivo, and caused no skin irritation upon histological examination. In a carrageenan-induced paw edema model in rats the topical application of developed bilosome gel significantly reduced paw swelling to 24.4% after 12 h, outperforming other treatments.

In another investigation, apigenin-loaded bilosomes exhibited spherical, nanosized vesicles (~210 nm to 430 nm), good PDI (<0.5), negative charge (−15 to −29 mV), and high encapsulation efficiency (~70% to 80%) [170]. The chitosan-coated optimized formulation (F2C1) resulted in an increased vesicle size (~300 nm), positive zeta potential (+17 mV), enhanced encapsulation efficiency (~90%), and improved drug release (~70%). The chitosan coating enhanced permeation and mucoadhesion (*p* < 0.05), as chitosan facilitates the opening of tight junctions, improving drug delivery. The formulation showed greater antimicrobial activity and higher efficacy against cancer cell lines, indicating its potential as an alternative to conventional delivery systems.

PEGylated bilosomes represent a novel approach by improving the stability, bioavailability, and therapeutic efficacy of poorly soluble phytochemicals. PEGylation provides a protective hydrophilic layer that prevents degradation, prolongs circulation time by reducing immune recognition, and enables controlled release of phytochemicals [171]. PEGylation also improves solubility, absorption, and targeting through passive and active mechanisms. Additionally, it enhances mucoadhesion and GI retention for oral delivery and supports better penetration for parenteral, topical, and ocular applications. Composed of lipids, surfactants, bile salts, and polyethylene glycol, this vesicle shares structural similarities with liposomes but offers additional benefits, including protection against enzymatic degradation in the GIT. They address limitations of traditional liposomes, such as encapsulation efficiency, stability, vesicle lysis, and scalability challenges. Bile salts like sodium deoxycholate serve as nontoxic, biocompatible solubilizing agents and are known to enhance permeation [172]. Additionally, PEGylated bilosomes provide high drug-loading capacity and enable controlled, extended drug release. Biochanin A-loaded PEGylated bilosomes was successfully formulated using the TFH method and optimized through Box–Behnken design [173]. The optimized Biochanin A-loaded PEGylated bilosomes demonstrated key properties (particle size; 216 ± 6.62 nm, EE; 80.54 ± 1.02%, PDI; 0.231, and zeta potential; −15.4 mV). The formulation exhibited prolonged drug release (88.23 ± 3.54% over 24 h) and high ex vivo intestinal permeation (56.97 ± 2.76% in 6 h). It achieved 4.70-fold higher bioavailability and significantly greater anti-inflammatory activity at all time points compared to pure drug dispersion.

Surface engineering of bilosomes is crucial to enhance their functionality and target specific delivery. Hyaluronic acid, a key extracellular matrix component and glycosaminoglycan in synovial fluid protects articular cartilage and regulates inflammation, cellular migration, and angiogenesis through various receptors. Hyaluronic acid-functionalized bilosomes have been developed for targeted delivery of tripterine, an anti-inflammatory phytomedicine, to inflamed joints via ligand–receptor interactions [164]. The formulation was developed using the TFH method with a cationic lipid and subsequent coating with hyaluronan. The optimized formulation was characterized by particle size (118.5 nm), EE (99.56%), and structural morphology. Studies on in vitro drug release, hemocompatibility, and cellular uptake confirmed excellent performance, with developed bilosomes achieving prolonged circulation time and a significant increase in intraarthritic bioavailability (799.9% compared to drug solution). In vivo evaluations demonstrated superior antiarthritic efficacy and inflammation resolution compared to uncoated vesicles.

A novel method for functionalizing phosphatidylcholine/cholesterol-based vesicular systems with sodium cholate hydrate (biosurfactant) and Pluronic P123 (triblock copolymer) was developed by [165]. Bilosomes were prepared using TFH combined with sonication, extrusion, and homogenization. Optimization studies evaluated nanocarrier size, charge, morphology, and stability, identifying a promising formulation for co-loading hydrophilic (methylene blue) and hydrophobic (curcumin) compounds. The high encapsulation efficiency highlights their potential for applications in drug delivery, anticancer therapies, and diagnostics. Another study effectively demonstrated the potential of bilosomes containing sulphated polysaccharide protein complexes of Enteromorpha intestinalis for oral therapy in hepatocellular carcinoma [163]. Treatment with optimized formulation (EH-Bilo-2) showed a substantial reduction in various biomarkers related to cancer, signifying the anticancer and antiangiogenic properties of developed bilosomes.

Photodynamic therapy, known for its high efficacy, safety, and noninvasive nature, has received significant interest as a therapeutic option for various cancer treatments. This approach is approved by the FDA for treating basal and squamous cell carcinomas as well as certain esophageal and lung cancers; it uses photosensitizing agents and laser radiation to generate reactive oxygen species (ROS) that kill cancer cells, damage tumor blood vessels, and activate immune responses. However, its effectiveness in metastatic cancers like melanoma is limited by defense mechanisms, melanin interference, and reduced photosensitizer activity [174]. Recent research has focused on plant-derived bioactive compounds, such as curcumin, as complementary agents in this minimally invasive treatment. These compounds enhance treatment by boosting ROS generation, inducing apoptosis, inhibiting angiogenesis and metastasis, suppressing cell proliferation, and modulating critical molecular pathways (e.g., MAPK, microRNA, p21). Self-assembled bilosomes loaded with curcumin, stabilized with L-α-phosphatidylcholine, sodium cholate, Pluronic^®^ P123, and cholesterol, were developed as a nanoplatform for photodynamic therapy [175]. These bilosomes co-encapsulated methylene blue (classical photosensitizer) and curcumin, as confirmed through spectroscopic techniques. In vitro studies demonstrated the stability, biocompatibility, and enhanced phototoxicity of the bilosomes’ formulation on melanoma (Me45) and epithelial (A375) cell lines, with selective uptake in tumor cells. Notably, the nanoplatform reduced Me45 melanoma cell viability to below 20% post-irradiation, outperforming non-encapsulated agents. Bilosomes encapsulating selected phytochemicals are presented in Table 5, along with details of their composition, preparation methods, and key characteristics.

### 3.6. Transferosomes

Transferosomes are ultra-deformable vesicular carriers composed of phospholipids, and edge activators (e.g., surfactants like Tween 80 or Span 80), which provide elasticity to the vesicles [184]. They are widely employed in transdermal drug delivery, particularly for systemic therapy and targeting deeper tissues. Unlike conventional liposomes, which are limited by the rigidity of their lipid bilayer, transferosomes incorporate edge activators that impart high flexibility, allowing them to traverse narrow intercellular spaces in the stratum corneum and penetrate via transfollicular routes [185]. This enhanced deformability contributes to improved skin permeation and deeper tissue penetration. Transferosomes are especially effective in delivering phytochemicals, as they enhance the stability, bioavailability, and permeability of these compounds while protecting them from degradation [186]. Additionally, they offer benefits, such as targeted delivery, sustained release, and suitability, for both hydrophilic and lipophilic drugs through a noninvasive route [187]. However, their application is associated with certain limitations, including formulation complexity, stability concerns, limited drug-loading capacity, and high production costs. Despite these challenges, transferosomes represent a promising strategy to improve the therapeutic efficacy of phytochemicals, particularly if scalability and stability issues are adequately addressed.

A recent study developed transferosomes incorporating *Rhodomyrtus tomentosa* leaf extract using L-α-phosphatidylcholine, cholesterol and sodium deoxycholate as edge activators [188]. Extract-loaded transferosomes formulated as a gel exhibited 81.90% EE, spherical vesicles (405.3 nm), low polydispersity index (0.16), and high zeta potential (−61.62 mV). They contained significant phenolic (15.65 μg gallic acid equivalent/g extract) and flavonoid (43.13 μg QE/g extract) levels. Extract-loaded transferosomes demonstrated greater antimicrobial activity (minimum inhibitory and bactericidal concentrations: 8–1024 μg/mL), strong antioxidant activity, and moderate tyrosinase inhibition (IC_50_: 245.32 μg/mL). Biocompatibility tests showed IC_50_ values of 7.05 and 4.73 μg/mL for L929 fibroblast and vero cells, respectively, and significantly reduced nitric oxide production by 6.78–88.25%. Stability studies confirmed durability under freeze-thaw conditions, with no significant changes in sedimentation or pH.

Transferosomes are often stabilized by incorporating hydrophilic polymers, which interact with the bilayer surfaces to create sterically stabilized vesicles in dispersion. Jabuticaba extract, rich in polyphenols, flavonoids, anthocyanins, and gallic acid, demonstrates strong antioxidant activity and promising biological efficacy [189]. The extract, incorporated into transferosomes modified with polymers (hydroxyethyl cellulose or sodium hyaluronate), demonstrated a larger particle size and greater storage stability compared to transferosomes without polymers [190]. The extract-loaded vesicles outperformed a reference solution in mitigating hydrogen peroxide toxicity and accelerating wound healing in cell monolayers, particularly with polymer enriched formulations. Table 6 presents transferosomes, ethosomes and transethosomes loaded with selected phytochemicals, providing details on their composition, preparation methods, and key features.

### 3.7. Ethosomes

Ethosomes, a third-generation vesicular system, are particularly notable due to their makeup with phospholipids and ethanol, which improves drug solubility and stability [215]. These structures consist of phospholipids (20–45%), water, and significant amounts of short-chain alcohols such as ethanol (20–45%) and isopropyl alcohol or propylene glycol (up to 15%) [216]. Their enhanced delivery capability, compared to vesicles like liposomes, is largely attributed to ethanol [216]. Ethanol, a known permeation enhancer, increases lipid fluidity and reduces the density of the lipid multilayer, a phenomenon called the ethanol effect. This is followed by the ethosome effect, where the nanovesicles penetrate and fuse with skin lipids, opening new pathways and releasing bioactive compounds into the skin’s deeper layers [215].

Pluronic F127 (Poloxamer 407), a nonionic triblock copolymer composed of a hydrophobic polypropylene oxide core flanked by hydrophilic polyethylene oxide segments, can self-assemble into micelles or form ordered aggregates depending on its concentration [217]. The modified novel plurethosomes, combining Pluronic F127 with phosphatidylcholine and ethanol, have been shown to significantly interact with lipid bilayers, aided by polypropylene oxide blocks, thereby enhancing the permeation of associated drugs. Mangiferin-loaded plurethosomes formed multilamellar vesicles that demonstrated particle sizes ranging from 200 to 550 nm, influenced by phosphatidylcholine concentration and the presence of polysorbate or Pluronic [218]. The biphasic release profile of plurethosomes highlighted their superior drug retention capability compared to unilamellar vesicles like transethosomes.

These carriers improve the delivery of both hydrophilic and lipophilic drugs without triggering adverse effects. Similarly, deformable vesicles, like transferosomes and transethosomes, incorporate penetration enhancers, enabling them to traverse biological barriers more effectively [22]. Despite their advantages, vesicular systems face challenges such as scalability, potential aggregation, and the need for precise formulation techniques to ensure long-term stability. However, their unique attributes, including targeted delivery, controlled drug release, and reduced systemic side effects, make them highly advantageous for various applications. These include the delivery of small molecules, proteins, vaccines, and even gene therapies, showcasing their versatility in addressing diverse therapeutic needs [219].

### 3.8. Transethosomes

Transethosomes are an advanced version of ethosomes, typically composed of ethanol, an edge activator (surfactant), phospholipids, and water, along with optional stabilizers (e.g., cholesterol), buffering agents, penetration enhancers, and imaging dyes, merging the properties of transferosomes and ethosomes [220]. Integrating surfactants with phosphatidylcholine in ethosome formulations enhances membrane flexibility and significantly improves their potential for transdermal delivery. This unique combination enhances biocompatibility, vesicle flexibility, and encapsulation efficiency compared to individual transferosomes and ethosomes [221]. Their particle size typically varies from 50 to 350 nm, mainly depending on the physicochemical characteristics of the drug [222].

Transethosomal gels were developed to overcome the low viscosity and retention of transethosomes, enhancing the skin delivery of herbal extracts and phytochemicals. Transethosomes gels loaded with extracts such as ginger, curcumin, and black cumin seed demonstrate enhanced antioxidant, anti-inflammatory, and anti-infective properties [223]. Recent research also highlights the incorporation of plant bioactives, like apigenin, glycyrrhizic acid, sinapic acid, hesperidin, hexatriacontane, mangiferin, catechin, and quercetin, into transethosome vesicles, resulting in improved bioavailability, stability, and solubility [223]. Transethosomes loaded with bioactive compounds from plants, such as *Carissa carandas* L. extract, or metabolites from plants, such as *Chenopodium murale* [208], have demonstrated effectiveness in therapeutic areas including antifungal, antibacterial, rheumatoid arthritis, antihyperlipidemic, anti-inflammatory, and cardiovascular treatments. These advancements highlight significant progress in transdermal delivery systems, offering promising applications for managing and treating various diseases [224]. Transethosomes offer a dual drug delivery system, enabling simultaneous transdermal and systemic administration, and are capable of encapsulating drugs with varying molecular weights. They address challenges like poor solubility, low bioavailability, and limited skin permeability, as seen in ginger extract [211] and catechins [225]. Their small particle size and elastic nature enhance skin penetration, making them effective for bioactive agents and large peptides, as demonstrated with palmitoyl pentapeptide [226]. This carrier has shown improvement in bioavailability, therapeutic efficacy, and patient compliance while minimizing the adverse effects of mangiferin [227]. However, challenges such as agglomeration, coagulation, and product loss during media transfer require careful preparation to ensure stability and efficiency.

Transethosomes overcome the stratum corneum barrier by leveraging their deformable and elastic vesicles, which allow them to penetrate deeply through the SC without rupturing, enhancing drug delivery in transdermal systems. Phospholipid ethanol interaction enhances drug permeation by disrupting the skin’s lipid bilayer and increasing fluidity, enabling flexible transethosome vesicles to penetrate deeper skin layers [224]. This carrier utilizes two primary mechanisms to enhance skin penetration: hydrotaxis and elastomechanics. Hydrotaxis drives the carrier along the skin’s hydration gradient, promoting lipid adhesion and membrane fusion for effective entry. Elastomechanics increases their flexibility upon dehydration, allowing the carrier to navigate tight skin junctions and penetrate deeply into the epidermis or dermis, improving drug delivery efficiency [228].

Encapsulation within transethosomes protects sensitive phytochemicals from environmental degradation, such as oxidation, light, and heat, resulting in improved stability and extended shelf life. The gel matrix complements this system by providing viscosity and bioadhesive properties, ensuring prolonged skin residence time and localized drug delivery while reducing systemic side effects [223]. Moreover, transethosomes can accommodate both hydrophilic and lipophilic phytochemicals, increasing formulation versatility. The non-greasy, cooling nature of gels further enhances patient compliance, making the formulation user-friendly. However, challenges exist in this approach. The formulation process utilizes typical methods, such as hot homogenization, cold methods, the thin-film lipid hydration method, and the mechanical dispersion technique, but may also involve advanced techniques like sonication or extrusion, which can increase production costs. Stability concerns may still arise due to potential interactions between phytochemicals, phospholipids, and gel excipients, affecting efficacy. Additionally, transethosomes have a limited drug-loading capacity, and certain excipients may irritate sensitive skin. Scaling up production to ensure consistent vesicle size and uniformity can also pose difficulties. Despite these limitations, careful optimization of materials and processes can maximize the benefits of transethosomal gel formulations for delivering phytochemicals effectively.

### 3.9. Cubosomes

Cubosomes are nanostructured lipid-based carriers with a cubic phase geometry, characterized by a highly ordered honeycomb-like internal structure formed through the self-assembly of amphiphilic lipids in water. Stabilization is achieved via electrostatic interactions or steric factors, leading to the development of nonlamellar phases [229]. Cubosomes are unique nanomaterials made from unsaturated monoglycerides like glycerol monooleate and phytantriol, forming bicontinuous cubic liquid crystalline structures [230]. Their lipid-rich core enables high loading of hydrophobic compounds, while their partially water-exposed surface allows effective delivery of hydrophilic molecules. Glycerol monooleate is widely used as a matrix for cubosome fabrication due to its nontoxic, biodegradable, and biocompatible properties [231]. This monoglyceride can form various structures upon contact with water, including reversed micellar phases and liquid crystalline phases (lamellar, reversed hexagonal, and cubic), depending on the preparation temperature and concentration [232]. Phytantriol is a stable, biocompatible, and biodegradable alternative to glycerol monooleate, offering higher resistance to esterase degradation [233]. It forms an inverse cubic structure in water at room temperature and transitions to a reverse hexagonal phase at elevated temperatures (40–60 °C), making it ideal for bicontinuous cubic crystalline phases.

Few studies have explored tailoring cubosome membranes for specific applications like drug delivery, imaging agents, and biosensors. One approach involves modifying the curvature of lipidic systems by introducing substances such as cholesterol (30 mol%), sucrose stearate (20 mol%), or octyl glucoside (10 mol%) to lipid bilayers, which alters the curvature elasticity, packing efficiency, and water channel diameter, potentially leading to phase transitions [234]. The incorporation of poloxamer into the cubosomes led to an increase in their complex viscosity, suggesting the formation of a tighter network structure that enhanced resistance to erosion in the prepared cubosomes [230].

Nanosized liquid lyotropic crystalline particles enable the loading of hydrophilic, lipophilic, and amphipathic compounds, offering enhanced solubility, stability, and bioavailability. Cubosomes play a critical role in delivering drugs by providing controlled and sustained release, enhancing cell permeation, improving absorption, and enabling targeted delivery through surface modifications [235]. In phytochemical delivery, cubosomes overcome challenges such as poor solubility, low stability, and limited bioavailability of plant-derived compounds. They enhance solubility, protect phytochemicals from degradation caused by environmental factors, and provide sustained release for prolonged therapeutic effects. Additionally, their biocompatibility and ability to transport across biological membranes make them highly effective for phytochemical delivery. However, cubosomes’ applications face challenges such as complex preparation methods, difficulties in scaling up production, sensitivity to environmental conditions, and potential cytotoxicity in certain formulations. Encapsulation efficiency can also vary depending on the physicochemical properties of specific phytochemicals. Despite these limitations, cubosomes hold significant potential as innovative carriers for phytochemical delivery, addressing key limitations of conventional formulations. The potential of this carrier in delivering capsaicin for topical and transdermal delivery was reported [236]. Two formulations were developed using glycerol monooleate and phytantriol and have shown sustained release as well as skin targeting. Another study reported the wound healing efficacy of β-sitosterol, a phytosterol, when formulated into cubosomes [237]. Additionally, the cubosome formulation of *Ruta graveolens* extract has demonstrated promising results in allergic asthma [238]. Table 7 summarizes various cubosome-loaded phytochemicals, highlighting their composition, preparation methods, and key features.

## 4. Preparation Methods

Phytosomes are prepared using various techniques that enhance the bioavailability and stability of plant-derived bioactive compounds. These methods include the rotary evaporator, anti-solvent precipitation, cosolvency, salting out, supercritical fluid, and freeze-drying, each with specific advantages and limitations. Conventional methods for liposome preparation include techniques such as gentle hydration of a phospholipid film, electroformation, coalescence of small vesicles, solvent injection, and detergent dialysis [246]. Other methods include reverse phase evaporation, the solvent spherule method, and size reduction of large liposome vesicles (e.g., ultrasonication, extrusion). In addition, there are novel methods such as microfluidic techniques (like micro hydrodynamic focusing, microfluidic droplets, pulsed jet flow microfluidics, lipid film hydration in microtubes), supercritical fluids, modified electroformation, freeze-drying of double emulsions, the membrane contactor method, hydration of phospholipids deposited on nanostructured materials, liposome formation by curvature tuning, and biomimetic reactions for vesicular self-assembly [247]. Vesicular nanocarriers are prepared using diverse techniques depending on their composition and intended application. Invasomes are typically produced by TFH followed by sonication or extrusion to enhance skin penetration [248]. Niosomes utilize methods like THF, ether injection, or microfluidization, forming bilayers from nonionic surfactants and cholesterol [249]. Bilosomes, incorporating bile salts for improved gastrointestinal stability, are prepared via TFH and further processed through sonication or extrusion [165,250]. Transferosomes involve TFH with edge activators and may use ultrasonic dispersion or ethanol injection to produce ultra-deformable vesicles [251]. Ethosomes, prepared by hot or cold methods with ethanol and phospholipids, often include sonication or extrusion to achieve optimal vesicle size [252]. Transethosomes combine features of ethosomes and transferosomes, using similar methods to enhance skin delivery [221,253]. Cubosomes are prepared either by dispersing bulk cubic phases (top-down) or by self-assembly from lipid solutions (bottom-up), typically stabilized with polymers like Pluronic F127 [229]. Table 8 provides a comprehensive overview of the preparation techniques, commonly used excipients, key features and stability characteristics of various vesicular nanocarriers used for the delivery of phytochemicals.

### 4.1. Thin Film Hydration

The TFH technique is one of the most widely used and classical methods for the preparation of vesicular carriers. In this method, lipids or surfactants and other excipients are first dissolved in a volatile organic solvent, typically chloroform or a chloroform–methanol mixture. The solvent is then evaporated under reduced pressure using a rotary evaporator, forming a thin, dry lipid film on the walls of a round-bottom flask. This film is subsequently hydrated with a buffered aqueous phase under continuous agitation, leading to the spontaneous formation of multilamellar vesicles. The resulting vesicles can be further processed using sonication or extrusion to achieve the desired size and lamellarity. This technique is appreciated for its simplicity, versatility, and ability to encapsulate both hydrophilic and lipophilic drugs. The phytosomes are prepared by mixing a stoichiometric ratio of phytochemicals and phospholipids in an appropriate organic solvent using the same procedure. TFH is a simple and widely used method but requires precise temperature control to prevent the degradation of sensitive phytochemicals. The solvent evaporation method was used to make complexes of oleanolic acid–phospholipid [254], while a rapid solvent evaporation method followed by a self-assembly technique was used to prepare berberine phospholipid complexes for the development of a berberine drug delivery system with higher efficiency [255]. Nano-phytosomes are phyto-phospholipid complexes, typically under 200 nm in size, designed to enhance absorption and biological activity. They have a composition similar to regular phytosomes or phyto-phospholipid complexes but offer improved delivery due to their nanoscale size. A stable colloidal phytosome delivery system was developed to enhance the antioxidant properties of pomegranate peel extract by utilizing phosphatidylcholine and gamma oryzanol through the TFH/sonication method [256]. The resulting homogeneous spherical nano-phytosomes at a ratio of 8:2:2% *w*/*w* (extract: phosphatidylcholine: gamma oryzanol) exhibited nano size (60.61 ± 0.81 nm), negative zeta potential (−32.24 ± 0.84 mV), acceptable polydispersity index (PDI, 0.19 ± 0.01), loading (19.13 ± 0.30%), and encapsulation (95.66 ± 1.52%). In a different study, the phytosome prepared using the TFH method with egg yolk phosphatidylcholine demonstrated greater encapsulation efficiency (98.27%) and better solubility (89.15%) [257]. It also exhibited good total phenolic content (114.44 mg gallic acid equivalent/g), ferric reducing antioxidant power (15.77 mmol Trolox/g), and radical scavenging activity (9.49 mmol Trolox/g). A phyto-phospholipid complex formulation encapsulating *Calendula officinalis* extract and gold nanoparticles was made using the TFH method with extrusion. The prepared vesicle with low particle size (under 100 nm) exhibited good encapsulation efficiency for both quercetin and chlorogenic acid [258]. They demonstrated notable wound healing and antioxidant activities compared to free components and plain liposomes. Cellular interactions were confirmed using fluorescence microscopy with Texas Red-labeled vesicles.

### 4.2. Anti-Solvent Precipitation Technique

The anti-solvent precipitation technique involves dissolving the phyto-phospholipid complex in a solvent, followed by precipitation with a nonsolvent, offering high encapsulation efficiency and cost-effectiveness. However, it may lead to incomplete precipitation and solvent residues that can affect product purity. Phytosome nanosuspensions for silybin–phospholipid complexes were successfully developed using the traditional solvent evaporation/antisolvent precipitation method to address potential formulation challenges of silybin such as low bioavailability due to poor solubility [259]. The complexes were prepared through the formation of weak intermolecular interactions between the polar head of the phospholipids and the hydroxyl group of silybin. In another study, hydroalcoholic Murraya koenigii extract and soy lecithin were dissolved in alcohol, while cholesterol was dissolved in dichloromethane [260]. The mixture was subsequently heated to 60 °C, concentrated, and hexane was added as an antisolvent to induce precipitation. Similarly, a phyto-phospholipid complex consisting of orange peel and liquorice extracts was refluxed with dichloromethane, followed by evaporation and precipitation with *n*-hexane [261]. Additionally, phytosomes incorporating *Bombax ceiba* extract [262] and *Allium cepa* phospholipid complexes [263] were prepared by this method.

### 4.3. Cosolvency Method

The cosolvency method is a widely used technique for preparing phyto-phospholipid complexes, involving the dissolution of plant extracts and phospholipids in compatible solvents, such as methanol, to prepare a uniform mixture. The extract is added dropwise to the phospholipid solution with continuous stirring to form the phytosomal complex [264]. This method is ideal for poorly soluble compounds and offers several advantages, including simplicity, cost-effectiveness, and the ability to achieve high encapsulation efficiency with controlled particle size. However, its limitations include the potential for solvent residues, the need for careful solvent selection to avoid degradation of sensitive compounds, and scalability challenges for large-scale production. A nano-phytosome in thermogel containing *Glycine max* (soybean) extract was developed and evaluated for its antiobesity effects on body weight, adipose tissue size, and lipid profile [78]. Three preparation methods, cosolvency, solvent evaporation, and salting out, were employed. The optimized formulation based on the cosolvency method demonstrated high encapsulation efficiency (>99%), particle sizes ranging from 51.66 to 650.67 nm, and drug release rates of 77.61% to 99.78%. Fourier-transform infrared and TEM analyses confirmed successful phytosome formation. In vivo studies showed significant reductions in body weight, adipose tissue mass, and lipid profile, highlighting its potential as an antiobesity treatment.

### 4.4. Salting-Out Method

The salting out technique involves adding a salt solution to reduce the solubility of the phyto-phospholipid complex, causing precipitation. It is cost-effective, minimizes organic solvent use, and is easy to scale, but may require optimization to avoid salt residue affecting purity. In a reported method, diosmin and soy phosphatidylcholine complexes were prepared using three methods: solvent evaporation, salting out, and lyophilization [265]. According to the solvent evaporation method, the drug and phospholipid were dissolved in a methanol–dioxane mixture, refluxed, and the solvent was evaporated; diosmin and lipids were added to a mixture of dimethyl sulfoxide, dehydrated ethanol, and chloroform, stirred until they were dissolved, and then precipitated with *n*-hexane.

### 4.5. Freeze-Drying Technique

The freeze-drying method involves freezing the phyto-phospholipid mixture and sublimating the solvent under vacuum to produce a dry phytosome powder, resulting in highly stable, lightweight, and porous formulations that are suitable for thermolabile compounds [266]. Using the lyophilization technique, both ingredients were mixed with dimethyl sulfoxide and t-butyl alcohol, stirred, and then lyophilized at −70 °C and −40 °C. The final complex had a yield of 90.4% and was stored in a desiccator at 4 °C. In another attempt, kaempferol phospholipid complexes were prepared using the lyophilization method [267].

### 4.6. Supercritical Fluid Technique

The supercritical fluid technique is an advanced method for preparing phytosomes, utilizing carbon dioxide under controlled conditions to produce thermally stable and high purity formulations with uniform particle sizes [62]. The process involves dissolving phytoconstituents and phospholipids in fluid, leading to phytosome formation upon controlled pressure or temperature reduction. Key advantages include ecofriendliness, enhanced bioavailability, and scalability, while limitations involve high costs, technical expertise, and safety concerns. Additionally, certain phytoconstituents may exhibit poor solubility in this fluid, necessitating the use of co-solvents that can complicate the process, and batch-to-batch consistency remains a challenge during scale-up. Development and evaluation of puerarin phospholipid complex microparticles using the supercritical fluids method have been described [268]. The effects of various processing variables, such as temperature, solution concentration, pressure, flow rate of supercritical carbon dioxide, and the drug solution on lipid complex particle characteristics were assessed. The complex obtained using this method consisted of amorphous, partially agglomerated spheres with particle sizes around 1 μm.

## 5. In Vitro and In Vivo Tests for Vesicular Carriers

In vitro tests simulate biological conditions to predict carrier behavior and optimize formulations before animal or human studies [269,270]. Table 9 presents frequently used in vitro and in vivo tests for vesicular carriers employed in various phytochemical delivery applications. Drug release studies, cell-based (Caco-2 cell permeability assay, parallel artificial membrane permeability assay), intestinal (Everted gut sac assay, Ussing chamber assay), blood–brain barrier (human brain microvascular endothelial cells (hCMEC/D3), skin based (Franz-diffusion cell assay), and endothelial cell-based (Electric cell-substrate impedance sensing) permeability tests, particle size, and zeta potential analyses assess release kinetics, absorption potential, stability, and colloidal properties. Additional tests, like antioxidant assays such as 2,2-Diphenyl-1-picrylhydrazyl (DPPH), 2,2′-Azino-bis(3-ethylbenzothiazoline-6-sulfonic acid (ABTS), Ferric Reducing Antioxidant Power (FRAP), Oxygen Radical Absorbance Capacity (ORAC), Ferrous Ion Chelating (FIC), Cupric Reducing Antioxidant Capacity (CUPRAC) and anti-inflammatory assays including enzyme-based (Cyclooxygenase inhibition assay, lipoxygenase inhibition assay, inducible nitric oxide synthase inhibition assay), cytokine-based (Tumor necrosis factor-alpha (TNF-α) inhibition assay, interleukin inhibition assays), cell-based (RAW 264.7 macrophage assay, peripheral blood mononuclear cell assay), oxidative stress-based (Reactive oxygen species (ROS) inhibition assay, lipid peroxidation assay), in vivo animal model (Carrageenan-induced paw edema test, cotton pellet granuloma assay, acetic acid-induced vascular permeability assay), and nuclear factor kappa B inhibition assay, measure the protective effects of phytochemicals, while photostability, thermal and oxidative stability tests evaluate degradation under environmental stress. Immunomodulatory cell-based (Lymphocyte proliferation, toll-like receptor activation), delayed-type hypersensitivity or Cyclophosphamide-induced immunosuppression animal models evaluate the ability of phytochemicals to either enhance or suppress the immune system. These in vitro evaluations provide crucial preliminary data on release, bioactivity, and stability. In vivo experiments, conversely, are performed using animal models to evaluate pharmacokinetics, distribution within the body, and effectiveness of treatments. Pharmacokinetic studies measure drug absorption, distribution, metabolism, and elimination through parameters like C_max_, T_max_, AUC, and half-life. Biodistribution studies track drug accumulation in target organs, while therapeutic efficacy tests evaluate specific biological effects, including anticancer, neuroprotective, and cardioprotective activities. Specialized tests, such as antiviral (cytopathic effect reduction assay, Quantitative PCR assay), antimicrobial (Kirby-Bauer test, time-kill kinetics assay, live/dead staining (SYTO9/PI), in vivo animal models (Murine sepsis model, wound infection model), and ex vivo and biofilm assays, antidiabetic (Streptozotocin (STZ) or Alloxan induced diabetic animal model), and hepatoprotective (Carbon tetrachloride or Paracetamol induced liver toxicity animal model), cardioprotective (Ischemia-reperfusion (I/R) injury animal model, myocardial infarction (MI) animal model), neuroprotective (Stroke, Alzheimer’s disease, Parkinson’s disease, traumatic brain injury animal model, Morris water maze test, au and β-Amyloid (Aβ) ELISA) provide insights into the carrier’s potential in specific disease models. Cell viability, necrosis and apoptosis tests such as 3-(4,5-Dimethylthiazol-2-yl)-2,5-diphenyltetrazolium bromide (MTT) assay, annexin V/PI staining, caspase-3/7, terminal dUTP nick end-labeling assays are widely used in phytochemical research to evaluate the biological activity, cytotoxicity, and therapeutic potential of plant-derived compounds. Together, these in vivo tests confirm the formulation’s safety, efficacy, and drug targeting capabilities. Overall, both types of evaluations are critical for ensuring that phytochemical-loaded vesicular carriers deliver therapeutic compounds effectively and maintain stability for optimal clinical outcomes.

## 6. Clinical Trials and Patents

Clinical trials and patents on phytochemicals emphasize their transformative role in modern drug delivery and therapeutic applications. In clinical research, various phytochemicals have demonstrated promising outcomes for conditions ranging from cancer to metabolic and cardiovascular diseases. For example, liposomal curcumin has progressed to Phase IIb trials (NCT02138955) for treating locally advanced metastatic cancer, aiming to improve pharmacokinetics and safety profiles through enhanced bioavailability. Similarly, ursolic acid (NCT02337933), betulinic acid (NCT03904511), and lutein (NCT01534533) are being investigated for metabolic syndrome, psychological stress, and carotid atherosclerosis, respectively, highlighting their potential for treating chronic and lifestyle-related disorders. Furthermore, compounds like thymoquinone and ginsenoside Rg3 have shown potential in reducing cancer recurrence and managing vascular dementia. Clinical trials on phytochemicals in vesicular carriers are limited, despite preclinical evidence supporting their potential to enhance bioavailability and therapeutic efficacy. The lack of registered trials highlights the need for further research to bridge the gap between preclinical findings and clinical applications.

Patents offer complementary insights by presenting innovations that optimize the delivery and therapeutic effectiveness of phytochemicals (Table 10). For instance, a recent invention features a brain-targeting liposome incorporating a paeonol-ozagrel conjugate disclosed in patent CN107823649B, designed to cross the BBB and provide prolonged drug circulation for cerebral conditions [295]. Another patent (CN105534907A) describes a macrophage membrane-based nano-delivery system with betulinic acid capable of inhibiting cancer proliferation by targeting specific cellular pathways. Other notable developments include liposomal systems for neurodegenerative diseases using echinacoside and synergistic cancer treatments combining thymoquinone and taxane. Additionally, formulations like allicin liposomes offer enhanced antimicrobial, protozoal, and tumor fighting capabilities, broadening the application scope of phytochemicals. Together these clinical and patented innovations underscore the strategic integration of phytochemicals in nanovesicular delivery systems, addressing critical issues like drug solubility, stability, and targeted delivery. These advances are poised to improve therapeutic efficacy, reduce side effects, and expand the range of diseases treatable by naturally derived compounds. This dual pathway of clinical validation and technological innovation fosters the translation of phytochemicals from research to practical, patient-centered treatments.

## 7. Future Perspectives, Challenges and Conclusions

The future of phytochemical delivery through various vesicular carriers discussed here offers immense potential for improving the therapeutic efficacy and bioavailability of plant-derived compounds. These carriers provide targeted drug delivery, enhanced solubility, and prolonged circulation times, addressing key limitations of conventional formulations. For instance, ethosomes and transethosomes have shown promise in transdermal delivery due to their flexibility and ability to penetrate deep into the skin. Liposomes, composed of lipid bilayer vesicles, can encapsulate both hydrophilic and lipophilic phytochemicals, enhancing solubility, stability, and controlled release [296]. Nonionic surfactant-based niosomes offer greater stability than liposomes, enhancing the transdermal and oral bioavailability of phytochemicals. Cubosomes, with their unique cubic structure, offer high drug-loading capacity and sustained release, making them suitable for chronic conditions [297]. Phytosomes, a specialized carrier, form complexes with phytochemicals such as flavonoids and polyphenols to enhance absorption and stability, particularly for poorly water-soluble compounds like curcumin and quercetin, which are widely used in nutraceutical formulations [186]. Similarly, nanophytosomes represent a recent advancement in lipid-based vesicles, characterized by their smaller size, designed to further enhance the delivery of plant-derived nutraceuticals. Nanotechnology improves the delivery and efficacy of phytotherapeutics and photosensitizers in photodynamic therapy by enabling their simultaneous encapsulation in biocompatible nanostructures. This approach overcomes pharmacokinetic challenges typically encountered in combination therapy, enhancing selective tumor accumulation and synergistic therapeutic effects through the enhanced permeability and retention effect [298]. Hybrid vesicular systems integrate different vesicle types to enhance the stability, encapsulation efficiency, and bioavailability of phytochemicals while overcoming individual limitations. A liposome–niosome hybrid system developed for curcumin demonstrated improved encapsulation, stability in biological fluids, and enhanced bioavailability compared to conventional vesicles [299]. Stimuli-responsive vesicles, which release phytochemicals in response to specific stimuli such as pH, temperature, or enzymes, are being investigated for targeted therapy [27]. Surface-modified vesicles functionalized with ligands such as folic acid and peptides are being investigated for the active targeting of phytochemicals to specific cells or tissues, enhancing therapeutic precision and efficacy [297]. Nano-vesicular carriers, such as liposomes, niosomes, and polymersomes, are designed to withstand gastric degradation and enhance intestinal absorption, improving the oral bioavailability of phytochemicals. Recent advancements, including chitosan-based nanoparticles, have further optimized solubility, stability, and bioactivity for more effective phytochemical delivery [33]. However, several challenges remain in optimizing these delivery systems. Stability during storage, large-scale production, and regulatory approval for these complex nanostructures pose significant hurdles. The encapsulation efficiency and controlled release of phytochemicals can also vary depending on the carrier type and preparation technique. Additionally, the high cost of some vesicular systems, along with the need for advanced characterization methods to ensure quality and reproducibility, can limit widespread applications. Low encapsulation efficiency in nanocarriers limits the therapeutic potential of phytochemicals in pharmacotherapy. Enhancing phytochemical loading through composition changes or layer-by-layer designs, along with surface modifications like ligand attachment or tumor-penetrating peptides, can improve targeting and penetration across biological barriers. Future research should focus on designing biomimetic, stimuli-responsive nanostructures to optimize targeted phytochemical delivery in disease treatment. Research must focus on developing scalable, cost-effective manufacturing processes and enhancing carrier stability while maintaining phytochemical efficacy. Collaborative efforts between research institutions and the pharmaceutical industry, alongside advancements in nanotechnology and regulatory frameworks, will be crucial to fully harness the therapeutic potential of phytochemicals through these innovative delivery platforms. Innovative preparation techniques, surface modifications, and hybrid systems play a crucial role in enhancing the clinical applicability of phytochemical delivery. Ongoing research and technological advancements will further optimize these nanocarriers, ensuring their continued relevance in treating chronic and complex diseases while improving patient outcomes.

## Figures and Tables

**Table 1:** Chemical structure, sources, activities, and formulation challenges of major phytochemicals.

Name	Structure	Botanical Sources	Type	Biological Role	Formulation Challenges
Allicin	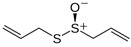	*Allium sativum*	Thiosulfinate	Antimicrobial, antioxidant, anti-inflammatory, cardioprotective, anticancer	Highly unstable, short shelf life, strong odor, low oral bioavailability
Artemisinin	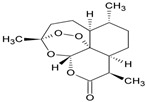	*Artemisia annua*	Sesquiterpene lactone	Antimalarial, anticancer, anti-inflammatory, antiviral, antiparasitic	Low solubility, short-half life, chemical instability, limited shelf life, drug resistance
β-carotene	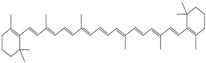	*Daucus carota*, *Ipomoea batatas*, *Cucurbita maxima*, *Spinacia oleracea*	Carotenoid	Provitamin A, antioxidant	Insoluble in water, prone to oxidation
β-Lapachone	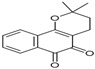	*Tabebuia avellanedae*	Quinone	Antioxidant, anti-inflammatory, cardioprotective, anticancer, antimicrobial	Poor solubility, low bioavailability, toxicity, short half-life
Betulinic acid	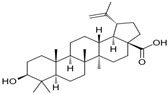	*Betula pendula*, *Syzygium formosanum*, *Ziziphus mauritiana*, *Diospyros melanoxylon*	Pentacyclic triterpenoid	Anti-cancer, anti-HIV, anti-inflammatory	Low water solubility, limited bioavailability
Canthaxanthin	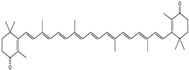	*Cantharellus cinnabarinus*, *Capsicum annuum*, *Daucus carota*	Keto-carotenoid	Antioxidant	Insoluble in water, prone to oxidation
Catechins	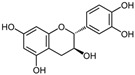	*Camellia sinensis*, *Theobroma cacao*, *Vitis vinifera*	Polyphenolic flavonoid	Antioxidant, anti-inflammatory, cardiovascular, anticancer, metabolic health, neuroprotective, antimicrobial	Poor solubility, low stability, rapid metabolism, low oral bioavailability,
Cinnamaldehyde	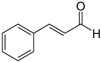	*Cinnamomum cassia*, *Cinnamomum verum*	Phenylpropanoid	Antimicrobial, antiparasitic, antioxidant, antidiabetic, anticancer, cardiovascular, neuroprotective	Volatility, low stability, poor solubility, mucosal irritation, rapid metabolism
Curcumin	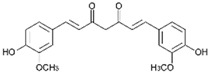	*Curcuma longa*	Diarylheptanoid	Anti-inflammatory, antioxidant, anti-cancer	Poor bioavailability, rapid metabolism
Echinacoside	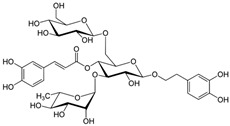	*Echinacea purpurea*, *Cistanche deserticola*, *Leonurus japonicus*	Phenylethanoid glycoside	Antioxidant, anti-inflammatory, neuroprotective, immunomodulatory, antiaging, wound healing, hepatoprotective	Low stability, poor solubility, low bioavailability, short half-life
Fisetin	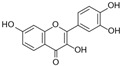	*Nelumbo nucifera*	Flavonoid	Antioxidant, anti-inflammatory, senolytic, neuroprotective, anti-cancer, cardioprotective	Poor solubility, sensitive to heat, oxygen and light, first-pass metabolism
Ginsenoside Rg3	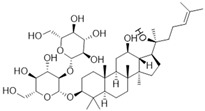	*Panax ginseng*, *Panax notoginseng*	Saponin	Anticancer, neuroprotective, anti-inflammatory, cardioprotective, antifatigue, immunomodulatory, antidiabetic	Poor solubility, low bioavailability, unstable, low permeability, first-pass metabolism
Honokiol	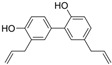	*Magnolia officinalis*, *Magnolia grandiflora*	Biphenol	Anticancer, neuroprotective, anti-inflammatory, antimicrobial, antiviral, cardioprotective, anxiolytic, antidepressant	Poor solubility, rapid metabolism, low stability, low intestinal permeability
Lutein	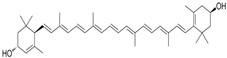	*Spinacia oleracea*, *Brassica oleracea*	Carotenoid	Antioxidant	Instability to light, poor solubility
Lycopene	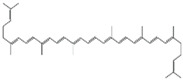	*Solanum lycopersicum* L., *Citrullus lanatus*, *Psidium guajava*	Acyclic carotenoid	Cardiovascular, reduce oxidative stress	Poor solubility
Paeonol	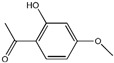	*Paeonia suffruticosa*, *Paeonia lactiflora*	Phenolic compound	Anti-inflammatory, analgesic, antioxidant, antimicrobial, immunomodulatory, cardioprotective, anticancer	Poor solubility, volatility, low stability, low bioavailability
Piperine	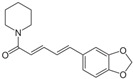	*Piper nigrum*, *Piper longum*	Alkaloid	Antioxidant, anti-inflammatory, antimicrobial, anti-carcinogenic, neuroprotective	Poor solubility, low bioavailability, light/heat sensitive
Plumbagin	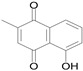	*Plumbago zeylanica*, *Diospyros lotus*, *Drosera rotundifolia*	Naphthoquinone	Anticancer, anti-inflammatory, antioxidant, cardioprotective, neuroprotective, antidiabetic, antimicrobial	Low stability, poor solubility, low oral bioavailability, short half-life, toxicity
Quercetin	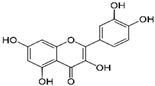	*Capparis spinosa*, *Malus domestica*, *Vaccinium corymbosum*	Flavonoid	Antioxidant, anti-inflammatory, anticancer, antiviral, antimicrobial, cardioprotective, neuroprotective	Poor solubility, low bioavailability, stability, low permeability
Resveratrol	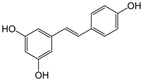	*Vitis vinifera*, *Arachis*, *Punica granatum*, *Polygonum cuspidatum*	Stilbene	Antioxidant, anti-inflammatory, anti-carcinogenic	Slightly soluble in water
Salvianolic acid B	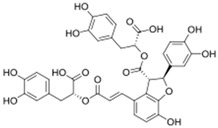	*Salvia miltiorrhiza*	Polyphenol	Antioxidant, anti-inflammatory, cardioprotective, neuroprotective, anti-fibrotic, anticancer, antidiabetic	Low oral bioavailability, instability, first-pass metabolism, short half-life, poor permeability
Thymoquinone	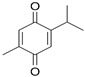	*Nigella sativa*	Benzoquinone	Hepato-protective, neuroprotective, nephroprotective, cardioprotective, anti-inflammatory, antimicrobial, anti-carcinogenic, antidiabetic, immunomodulatory	Poor solubility, instability, low bioavailability, bitter taste
Ursolic acid	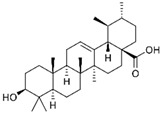	*Rosmarinus officinalis*, *Prunella vulgaris*, *Ocimum sanctum*	Triperpenoid	Antioxidant, anti-inflammatory, anti-cancer, antimicrobial, cardioprotective	Poor solubility, low bioavailability, unstable
Verbascoside	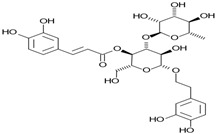	*Verbascum thapsus*	Phenylethanoid glycoside	Antioxidant, anti-inflammatory, neuroprotective, hepatoprotective, antimicrobial, nephroprotective, wound healing, anticancer	Low stability, poor solubility, low bioavailability, short half-life
Wogonin	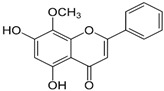	*Scutellaria baicalensis*	Flavonoid	Anticancer activity, neuroprotective, anti-inflammatory, antiviral	Poor solubility, low stability, first-pass metabolism

**Table 2:** Comparison of advantages, limitations, and key attributes of nanovesicular carriers.

Carrier	Advantages	Limitations	Skin Permeation	Entrapment Efficiency	Drug Loading	Scalability	Stability	Cost
Phytosomes	Enhanced bioavailability, natural origin, suitable for phytoactives	Limited to amphiphilic/lipophilic phytochemicals, cost of phospholipid	Moderate	High	Moderate to high	Good, relatively simple formulation methods	Moderate, sensitive to oxidation	High due to phospholipids
Liposomes	Biocompatible, versatile, enhanced drug delivery	Low stability, high cost, potential leakage	Moderate, depending on formulation	Moderate to high	Moderate	Challenging due to cost and stability issues	Low to moderate, prone to oxidation	High due to phospholipids
Invasomes	Enhanced active delivery through stratum corneum	Volatility of terpenes, moderate stability	High	Moderate to high	Moderate	Moderate, due to terpene handling	Moderate, sensitive to terpene loss	Moderate, terpene inclusion increases cost slightly
Niosomes	Stable, cost-effective, customizable	Less biocompatible compared to liposomes, may require specific surfactants	Moderate to high, dependent on surfactant type	High	Moderate to high	Good, cost-effective methods available	Good, more stable than liposomes	Low to moderate, cost-effective materials
Bilosomes	Enhanced oral bioavailability, protects against gastric degradation, stable under physiological conditions	Limited skin delivery application, bile salts may interact with other excipients	Low to moderate	High	Moderate to high	Good, suitable for large-scale production	High, stable in GI tract and physiological pH	Low to moderate, bile salts are cost-effective
Transferosomes	High deformability, deep skin penetration	Requires special handling, potential instability	High due to deformability	Moderate to high	Moderate	Moderate, requires specific techniques	Moderate, edge activators may affect stability	Moderate, depends on edge activators
Ethosomes	Improved skin permeation due to ethanol, effective for transdermal delivery	Ethanol may irritate, stability issues	High due to ethanol’s effect on skin lipids	High	Moderate to high	Moderate to high, depends on ethanol handling	Moderate, ethanol can evaporate over time	Moderate, ethanol adds to cost
Transethosomes	Combines ethosome and transferosome benefits, superior penetration	Complex composition requires precise formulation	Very high, superior to ethosomes and transferosomes	Very high	High	Moderate, due to complexity	Moderate, requires optimized storage	Moderate to high, complex formulation increases cost
Cubosomes	High stability, controlled and sustained drug release, large surface area, high drug-loading capacity	Complex preparation process, potential viscosity issues, limited scalability	Moderate to high, depends on formulation	High, due to large internal surface area and cubic structure	High, suitable for both hydrophilic and lipophilic drugs	Moderate, requires specialized techniques like high pressure homogenization	High, resistant to degradation, but sensitive to temperature and pH fluctuations	Moderate, influenced by surfactant and stabilizer costs

**Table 3:** Phytochemical name, preparation technique, composition, and highlights of liposomal delivery of phytochemicals.

Phytochemical	Preparation Technique	Composition	Disease/Therapeutic Application	Highlights	Ref.
β-carotene and lutein	Thin film hydration with ultrasonication	Dipalmitoyl-phosphatidylcholine, L-α-phosphatidyl-ethanolamine, L-α-cephalin (3-snphosphatidyl-ethanolamine), stearylamine	Antioxidants, cancer, cardiovascular, skin diseases, eye health, anti-inflammatory effects	β-carotene adheres to the liposomal boundary, causing external morphological changes. Encapsulation efficiencies of lutein and β-carotene are over 98.8% and 87%, respectively. Lutein in cationic liposomes demonstrates better in vitro release stability (30%) compared to β-carotene (45%) between 3 and 6 h, with a lower leakage rate ensuring higher lutein retention.	[102]
β-lapachone	Thin film hydration	Soybean phosphatidylcholine, 1,2-distearoyl-*sn*-glycero-3-phosphoethanol amine-[*N*-(Carbonyl-methoxypolyethylene glycol-2000), cholesterol	Breast cancer	Uncoated and concanavalin A (ConA) coated β-lapachone liposomes exhibit nano size (100–150 nm). Hemagglutination assays verified ConA avidity, showing that Lipo-ConA and Lipo-PEG-ConA could hemagglutinate red blood cells. Site-specific liposomes demonstrated enhanced toxicity. ConA-coated liposomes achieved greater internalization in MCF7 cells compared to uncoated liposomes.	[103]
Allicin	Reverse phase evaporation	Lecithin, cholesterol	Diarrhea, flatulence, edema, arthritis, worm infestation and pulmonary complaints	Optimized allicin liposomes showed higher EE, low vesicle size and good stability. The formulation demonstrated sustained release behavior, highlighting their potential for controlled release for wider applications.	[104]
Artemisinin	Thin film hydration	Soy lecithin, cholesterol,1,2-distearoyl-*sn*-glycero-3-phosphoethanolamine-*N*-(polyethylene glycol)-2000	Antimalarial	Artemisinin-loaded liposomes, modified with a reversibly activatable cell penetrating peptide ((HE)10-G5-R6 or HE-R6), demonstrated sustained release, enhanced internalization, and increased cytotoxicity compared to non-modified liposomes. Additionally, modified liposomes exhibited prolonged tumor retention and improved tumor suppression.	[105]
Betulin and betulinic acid	Lipid hydration with extrusion	Dry egg-phosphatidylcholine/dipalmitoyl-phosphatidylcholine	Cytotoxic properties	Betulin and betulinic acid influence mitochondrial bioenergetics and membrane behavior by inhibiting oxidative phosphorylation, affecting H_2_O_2_ production, and inducing mitochondrial and liposome aggregation. Laurdan fluorescence studies reveal that these compounds cause phase heterogeneity in lipid systems, linked to their interaction with lipid bilayers.	[106]
Canthaxanthin	Hydration with sonication	Soy lecithin	Photoprotectors, antioxidants, anti-inflammatory, immunity enhancer and reproductive health.	Canthaxanthin-loaded vesicles (63–87 nm) were stable, biocompatible, and effective in protecting skin cells. Vesicles preserved fibroblast viability, reduced oxidative and inflammatory responses, and promoted cell migration, showcasing the potential for skin protection and regeneration through their antioxidant, anti-inflammatory, and antiaging effects.	[107]
Catechins and curcumin	Microfluidic	Dipalmitoylphosphatidylcholine, cholesterol	Tumor	Dual-loaded liposomes with curcumin and catechin exhibited nanoscale size (<200 nm) with encapsulation efficiencies of 100% for curcumin and 16.77% for catechin. While curcumin, catechin, and liposomes individually enhanced antiproliferation activity in colon cancer cells, the dual-loaded liposomes showed significantly greater inhibitory effects (*p* < 0.05). The results highlight the potential of dual encapsulation.	[108]
Cinnamaldehyde	Ethanol injection	Egg yolk lecithin, tween 80	Antibacterial	Higher cinnamaldehyde loading reduced liposome membrane fluidity. Antibacterial studies revealed that liposome-encapsulated cinnamaldehyde retained its ability to inhibit *Staphylococcus aureus* by disrupting cell membrane integrity, demonstrating greater persistence than pure cinnamaldehyde.	[109]
Curcumin	Film hydration with hand extrusion	1,2-dioleoyl-*sn*-glycero-3-phosphocholine, 1,2-dioleoyl-*sn*-glycero-3-phosphoethanolamine-*N*-[methoxy(polyethylene glycol)-2000], cholesterol	Anticancer	Liposomes loaded with curcumin demonstrated low particle size and excellent physical stability. Dilution showed rapid partitioning to the lipid bilayer. The in vivo efficacy of curcumin is unlikely to improve with liposomes, as cytotoxicity and uptake studies revealed reduced effectiveness in the 3D cell model.	[85]
Echinacoside	Thin film hydration	Soybean phosphatidylcholine, cholesterol, peptide angiopep-2, DSPE-PEG2K-MAL, DSPE-PEG 2K	Parkinson’s disease	Peptide angiopep-2 modified PEGylated echinacoside-loaded liposomes showed significantly higher brain uptake than others. It also showed superior efficacy in reducing MPTP-induced behavioral impairments, oxidative stress, dopamine depletion, and dopaminergic neuron death. This formulation significantly enhanced the neuroprotective effects of drug in the Parkinson’s disease model.	[110]
Epigallocatechin gallate and quercetin	Thin film hydration	Lecithin, cholesterol, tween 80	Antioxidant	Liposomes showed nanosized with encapsulation efficiencies of 64.05% for epigallocatechin gallate and 61.73% for quercetin. After 30 days, size increased slightly by 4.05%, with no significant changes in stability. The DPPH assay confirmed a synergistic antioxidant effect of the encapsulated compounds.	[111]
Fisetin	Film hydration	Dioleyl-phosphatidylcholine, cholesterol, 2-dioctadecylcarbamoyl-methoxyacetylamino) acetic Acid-(Ω-methoxy)-polyethylene glycol 2000 ester	Glioblastoma	The formulation exhibited a particle size of 173 ± 8 nm, with drug loadings of 1.7 ± 0.3% for fisetin and 0.8 ± 0.1% for cisplatin, and demonstrated stability over time. Encapsulation preserved the antiangiogenic activity of fisetin, and the formulation displayed an additive therapeutic effect of fisetin and cisplatin on GBM cells.	[112]
Genistein and plumbagin	Lipid film	L-α-phosphatidylcholine, 1,2-dipalmitoyl-*sn*-glycero-3-phosphoethanolamine-*N*-[methoxy(polyethylene glycol)-2000] ammonium salt	Prostate tumor	Nanoliposomes (~100 nm) encapsulating plumbagin and genistein demonstrated ~80% inhibition of prostate tumor growth in xenograft models without significant toxicity. The formulation inhibited the PI3K/AKT3 signaling pathway and reduced Glut-1 transporter levels, slowing tumor growth. The observed antitumor effects were linked to reduced cell proliferation and decreased blood vessel formation.	[113]
Honokiol	Thin film hydration with sonication	Soybean phospholipids, cholesterol, DSPE-mpeg2000	Osteosarcoma	Hyaluronic acid phospholipid conjugates were used to combine honokiol-loaded liposomes which have low particle size and high encapsulation efficiency. The developed formulation inhibited cell proliferation, induced apoptosis, blocked the cell cycle and disrupted mitochondrial activity.	[114]
Lycopene	Thin film hydration	Soybean phosphatidylcholine, cholesterol	Antitumor	Lycopene-loaded liposomes possess low particle size. In vitro, the combination of lycopene and doxorubicin significantly increased cytotoxicity, while in vivo it reduced tumor size in B16 melanoma bearing mice and alleviated doxorubicin induced cardiotoxicity.	[115]
Paeonol	Film dispersion	Lecithin, cholesterol, polyethylene glycol-*p*-nitrophenyl ester, dioleoyl-phosphatidyl-ethanolamine	Hypertrophic scars	The particle size of prepared formulation was 235.7 nm. They significantly lowered scar proliferation, improved collagen structure, and reduced VEGF, TGF-β1, and TNF-α levels, demonstrating strong therapeutic potential for scar treatment.	[116]
Piperine	Thin layer dispersion	Cholesterol, phosphatidylcholine, sodium cholate, chitosan	Breast cancer	The drug included liposomes and chitosan-coated drug-loaded liposomes had nanometric sizes (165.7 to 243.4 nm). A biphasic release, improved permeation, and better in vitro antioxidant activity were noticed with liposomes when compared to piperine. The anticancer study with selected liposomes showed significant reduction of IC_50_ (*p* < 0.001) compared to pure drug.	[117]
Podophyllotoxin	Thin layer dispersion	Lecithin, cholesterol	Prostate cancer	In vitro drug release showed a gradual, time-dependent release pattern, with a cumulative release of 70.3% after 24 h. Cell viability tests on PC3 cells indicated that PPT-Lips had more effective anticancer activity than free PPT.	[118]
Quercetin	Thin film hydration	Soybean lecithin, cholesterol, polyethylene glycol 4000	Diabetic nephropathy	The antidiabetic effects of liposomes were demonstrated in rats with diabetic nephropathy. Liposomes and free drug improved biochemical and pathological markers of nephropathy. Liposomes showed higher drug plasma than pure drug.	[119]
Resveratrol and curcumin	Thin film evaporation with ultrasonication	Egg yolk phosphatidylcholine, tween 80	Antioxidant	The liposome formulation co-loading curcumin and resveratrol in a 5:1 ratio exhibited small particle size. It demonstrated superior antioxidant properties and better stability compared to liposomes loaded with individual polyphenols.	[120]
Salvianolic acid B		Soybean phospholipid, hydrogenated soybean phosphatidylcholine, 1,2-dipalmitoyl-*sn*-glycero-3-phosphocholine, 1,2-dipalmitoyl-*sn*-glycero-3-phospho-(1-rac-glycerol)	Idiopathic pulmonary fibrosis	Liposomes made of saturated neutral and anionic phospholipids have higher stability and permeability compared to unsaturated or cationic phospholipids. Better efficacy was observed in bleomycin induced idiopathic pulmonary fibrosis mice model when inhaled salvianolic acid B-loaded liposomes, attributed to inflammation inhibition and regulation of the coagulation fibrinolytic system.	[121]
Thymoquinone	Thin layer evaporation with ultrasonication	Egg phosphatidylcholine, phospholipon 90 G, plurol oleique, sodium hyaluronate	Dry eye disease	Drug-loaded liposomes reduced proinflammatory markers (Interleukin-1β, Interleukin-6, and tumor necrosis factor) and mitochondrial reactive oxygen species. These findings demonstrated the developed liposomes can enhance the efficacy of thymoquinone in treating dry eye disease.	[122]
Ursolic acid and ginsenoside Rg3	Reverse evaporation and pH gradient	Lecithin, cholesterol and (1,2-distearoyl-*sn*-glycero-3-phosphoethanolamine-*N*-[poly(ethylene glycol)]-2000) with glycyrrhetinic acid	Hepatocellular carcinoma	Liposomes containing ursolic acid and ginsenoside Rg3 showed good EE, drug loading, and sustained drug release. Cell experiments confirmed that codelivery of these actives significantly reduced cell viability, increased apoptosis, and elevated the proportion of cells.	[123]
Verbascoside	Film hydration	Soy phosphatidylcholine, cholesterol	Antineuropathic activity	Prepared liposomes have low particle size and sustained drug release (~82.28% in 24 h). In chronic constriction injury rat models, the liposomal formulation (100 mg/kg, i.p.) provided an extended antihyperalgesic effect compared to drug solution, with effects appearing within 15 min and lasting up to 60 min.	[124]
Vincristine	Ethanol injection with extrusion	Sphingomyelin, cholesterol, 1, 2-distearoyl-*sn*-glycero-3-phosphoethanolamine-*N*-[methoxy (polyethylene glycol)-2000]	Hematological malignancies and solid tumors	Liposomes with drug lipid ratio (1:5) showed reduced degradation (2.9% in 12 months at 2–8 °C) and extended in vivo half-life (22.7 h). This formulation demonstrated lower toxicity and better antitumor efficacy in a human melanoma model.	[125]
Wogonin	Reverse evaporation	Soybean phospholipids, cholesterol, 3-succinyl 30-stearyl glycyrrhetinic acid	Liver cancer	Three types of formulations were tested for antitumor efficacy: drug solution, liposomes, and glycyrrhetinic acid-modified liposomes. The modified liposomes exhibit greater entrapment, cellular uptake, liver accumulation, prolonged retention, and anticancer effects compared to others.	[126]

**Table 4:** Phytochemical name, preparation technique, composition, and highlights of niosomal delivery of phytochemicals.

Phytochemical	Preparation Technique	Composition	Disease/ Therapeutic Application	Highlights	Ref.
Amygdalin	Thin film hydration	Dihexadecyl phosphate, tween 60, cholesterol	Alzheimer’s disease	Nanocarrier enhanced working memory and recognition, reduced oxidative stress, and restored brain monoamine and neurotransmitter levels. Gene expression analysis revealed significant downregulation of BAX, with upregulation of BCL2, acetylcholinesterase, and monoamine oxidase.	[144]
Artemisinin and metformin	Thin film hydration	Cholesterol, span 60	Lung cancer	PEGylated magnetic niosomes developed facilitated rapid tumor-targeted drug delivery. In vitro studies on A549 lung cancer cells showed significant toxicity under a magnetic field, increased pro-apoptotic Bax expression, decreased antiapoptotic Bcl2 expression, and effective cellular uptake via endocytosis.	[143]
β-carotene	Thin film hydration	Spans 40, 60, 80, tween 20, 40, 60, cholesterol	Cancer, cardiovascular diseases, arteriosclerosis, and macular degeneration	The developed formulation has good stability against sunlight, temperatures, oxidative stress, and was stable in culture medium. It was effectively taken up by immortalized and transformed cells at concentrations of 0.1–2 μM, suggesting the formulation as an efficient method for β carotene delivery.	[145]
Canthaxanthin	Thin film hydration with sonication	Span 60, cholesterol	Antioxidant and free radical scavenging	Niosomes prepared with span 60 and cholesterol at a 1:1 molar ratio showed smaller, uniform sizes with higher EE. Stability tests indicated higher degradation under light and higher temperatures. The addition of cholesterol significantly improved drug stability.	[146]
Catechin	Thin film hydration	Tween 60, lauryl alcohol, cetyl alcohol, cholesterol	Antioxidant	Developed carrier has nanosize, narrow size distribution, good EE (85.82%), and sustained catechin release. Milk fortified with niosomes maintained its sensory and physicochemical properties while exhibiting enhanced antioxidant activity.	[147]
Curcumin	Thin film hydration with ultrasonication	Span 60, cholesterol	Anticancer	Developed niosomes showed high entrapment (~99.8%), loading (68.33%), and sustained release (~25.49 in 336 h). The formulation was biocompatible and exhibited dose-dependent toxicity against cancer cells (IC_50_ at 200 µg/mL, containing 66.75 µg drug).	[148]
Diosgenin	Thin film hydration	Span 40, tween 40, cholesterol	Anticancer	Diosgenin niosomes exhibited nanosize, higher loading efficiency (~89%), controlled and sustained drug release. The viability of HepG2 cell line treated with free diosgenin was 61.25%, which decreased to 28.32% upon niosomes, significantly enhancing its anticancer efficacy.	[149]
Epigallocatechin-3-gallate	Thin film hydration	Span-60, tween-60, cholesterol, DSPE-PEG (2000)-carboxylic acid	Lung cancer	Prepared niosomes showed ideal pharmaceutical characteristics. In vitro studies of cetuximab conjugated to niosomes on A549 lung carcinoma and BEAS-2B normal bronchial cells assessed cytotoxicity, apoptosis, fluorescence imaging, and theranostic potential.	[150]
*Fumaria officinalis* active rich fraction	Ether injection	Span 60, brij 52, cholesterol	Antidiabetic, anti-neuropathic, anti-inflammatory	Optimized formulation (Nio-2) with stylopine (48.3%) and sanguinarine (51.6%) demonstrated high entrapment and stability. *Fumaria officinalis*, active rich fraction, and Nio-2 showed significant antidiabetic and anti-inflammatory activity, hence suggested as an alternative therapy in inflammations and neuropathic pain.	[140]
Lycopene	Microfluidic and thin film hydration with probe sonication	Span 60, cholesterol	UVB protection and anti-hyperpigmentation	Niosomes displayed good uniformity, nano size, and low PDI when processed through a hydrodynamic flow-focusing platform. The drug release showed Korsmeyer–Peppas kinetics, and the formulation showed enhanced stability, strong UVB protection, and anti-melanogenesis effects.	[151]
Paeonol	Modified ethanol injection	Span 60, cholesterol, polyethylene glycol monostearate	Anticancer	Optimized PEGylated niosomes exhibited nano size, good entrapment, and sustained release. Pharmacokinetic studies in rats showed a higher elimination half-life (87.5 vs. 17.0 min) and a higher AUC (38.0 vs. 19.48 μg/mL·min) compared to the free drug. Enhanced cytotoxicity was observed against HepG2 cells, with IC_50_ values of 22.47 μg/mL.	[152]
Piperine and curcumin	Thin film hydration	Span 60, cholesterol	Antioxidant, Protection against Paraquat-induced pulmonary toxicity	Optimized niosomes demonstrated high encapsulation efficiency (>85%), nanosize (264–286 nm), a narrow PDI (0.19–0.23), and stability for 90 days. Co-treatment with curcumin and piperine-loaded nanoparticles effectively restore mitochondrial function, hence reduce acute pulmonary toxicity.	[153]
Plumbagin	Solvent evaporation	Span 20, cholesterol	Antidiabetic	The optimized niosomes demonstrated antioxidant activity and enzyme inhibition of α-amylase (90.69%) and α-glucosidase (88.43%). The data in this study show significant potential for diabetes management by inhibiting oxygen radicals and key enzymes involved in glucose metabolism by the developed carrier.	[154]
Quercetin	Hydration with high pressure homogenization	Span 60, creemophor RH40	Skin whitening and antioxidant	The best formulation was prepared with span 60 Cremophor RH40 ratio of 9:11. Niosomes enhanced quercetin’s solubility and photostability while offering sustained release and superior transdermal penetration. Skin retention of quercetin niosomes was 2.95 times higher than that of quercetin solution.	[155]
Thymoquinone	Solvent evaporation and hydration with sonication	Span 20, 40, 60, tween 20, 40, 80, cholesterol, soya lecithin	Anticancer, antioxidant, anti-inflammatory, and antimicrobial	Optimized niosomes (TMQNIOS) demonstrated low vesicle size (~157.32 to 211.44 nm) and encapsulation (~60–80%). Drug release followed Higuchi’s release kinetics and greater permeation across intestinal mucosa from developed carriers.	[156]
Thymoquinone and carum extract	Thin film hydration	Span 60, tween 60, ergosterol	Breast cancer	Two formulations, namely Nio/TQ (thymoquinone) and Nio/Carum (carum extract), were developed. The MTT assay showed these carriers have greater anticancer activity than their pure form against MCF-7 cancer cell line. Cell cycle analysis revealed G2/M arrest in the TQ, Nio/TQ, and Nio/Carum formulations.	[157]
Vincristine	Thin film hydration	Span 60, tween 60, cholesterol-PEG 600: diacetyl phosphate	Lung cancer	PEGylated drug-containing niosomes demonstrated significantly higher cytotoxicity against TC-1 lung cancer cells and stronger tumor inhibitory effects in lung tumor bearing C57BL/6 mice compared to free drug, suggesting its potential as a promising delivery system for vinblastine in cancer treatment.	[158]

**Table 5:** Phytochemical name, preparation technique, composition, and highlights of bilosomal delivery of phytochemicals.

Phytochemical	Preparation Technique	Composition	Disease/ Therapeutic Potential	Highlights	Ref.
Enteromorpha intestinalis-sulfated polysaccharide-protein complexes	Thin film hydration	Sodium cholate, span 65	Hepatocellular carcinoma	Optimized formulation consisted of spherical vesicles (181.1 ± 16.80 nm) with moderate drug entrapment efficiency (71.60 ± 0.25%) and exhibited controlled release behavior. In disease-induced rats, treatment with bilosomes significantly reduced serum levels of α-fetoprotein, endoglin, lipocalin-2, and heat shock protein 70 compared to untreated rats. Liver histology revealed focal degeneration of pleomorphic hepatocytes with mild fibrosis extending from the portal area.	[163]
Tripterine	Thin film hydration	Soybean phosphatidylcholine,1, 2-stearoyl-3-trimethylammonium-propane, Sodium deoxycholate, hyaluronic acid	Arthritis	Hyaluronic acid-coated tri-loaded bilosomes had a particle size of 118.5 nm and high entrapment efficiency (99.56%). Demonstrated excellent cellular uptake and significantly enhanced intra-articular bioavailability—799.9% > Tri solution. In arthritic animal models, coated tri-loaded bilosomes showed markedly improved pharmacokinetics and superior antiarthritic efficacy compared to uncoated Tri-BLs, leading to notable inflammation reduction.	[164]
Berberine	Thin film hydration	Soybean lecithin, sodium deoxycholate, cholesterol, chitosan, carbopol 974 NF	Rheumatoid arthritis	Optimized chitosan-coated, berberine-loaded bilosomes had a mean particle size of 202.3 nm, entrapment efficiency of 83.8%, and a surface charge of +30.8 mV. Demonstrated a sustained in vitro release profile, enhanced ex vivo skin permeability and confirmed non-irritant nature via histological studies. Topical application of the berberine-loaded bilosomal gel significantly reduced inflammation in a rat model of carrageenan-induced paw edema.	[169]
Apigenin	Solvent evaporation method	Cholesterol, sodium deoxy cholate, tween 80, phosphatidylcholine, chitosan	Antimicrobial, anticarcinogenic	Optimized chitosan-coated bilosome formulation showed an increased vesicle size (298 ± 3.56 nm), positive surface charge (+17 mV), high encapsulation efficiency (88.1 ± 1.48%), and enhanced drug release (69.37 ± 1.34%). TEM analysis revealed smooth, non-aggregated vesicles. Antimicrobial and cytotoxicity studies confirmed its superior efficacy, displaying larger inhibition zones and greater activity against two cancer cell lines.	[170]
Biochanin A	Thin film hydration	Sodium deoxy cholate, span 60, polyethylene glycol-2000SA	Anti-inflammatory	Selected PEGylated bilosome formulation displayed a vesicle size of 216 ± 6.62 nm, entrapment efficiency of 80.54 ± 1.02%, polydispersity index of 0.231, and zeta potential of –15.4 mV. X-ray diffraction confirmed successful drug encapsulation within the bilosomal matrix. It showed a sustained release profile (88.23 ± 3.54% over 24 h) and high ex vivo intestinal permeation (56.97 ± 2.76% in 6 h). Compared to pure Biochanin A dispersion, the optimized Pegylated formulation demonstrated 4.7-fold greater bioavailability and enhanced anti-inflammatory activity.	[173]
Curcumin	Thin film hydration method	L-α-phosphatidylcholine, sodium cholate, pluronic^®^ P123, and cholesterol	Melanoma	PEGylated bilosomes demonstrated protective effects and enhanced cellular uptake in A375, Me45, and HaCaT cell lines. They showed selective targeting of tumor cells, with significantly greater cytotoxicity against Me45 melanoma cells, reducing their viability to below 20% after 24 h of phyto-photodynamic treatment.	[175]
Costunolide	Thin film hydration	Span 85, cholesterol, bile salt	Colon cancer	Formulation exhibited superior cytotoxicity against LS174T colon cancer cells and demonstrated safety and selectivity in normal colonic epithelial cells, as compared to raw drug. Bilosomes were found to be more effective in up-regulating proapoptotic genes, down-regulating antiapoptotic BCL2 mRNA, enhancing cytochrome C release, increasing reactive oxygen species generation, and disrupting mitochondrial membrane integrity.	[176]
Curcumin	Thin film hydration	Span 60, sodium deoxycholate, cholesterol	Hepatoprotective and renal protective effect	The formulation showed ideal pharmaceutical characteristics, drug release, good stability, and reduced cytotoxicity compared to free drug. The formulation demonstrated effective hepatic and renal protection in liver cirrhosis-induced rats, preserving organ function and histological integrity.	[177]
Epigallocatechin gallate	Ethanol injection	Tween 40, cholesterol	Antioxidant, anti-cancer, regulation of fatty acid metabolism, improvement of intestinal immunity	Bilosomes demonstrated superior gastrointestinal stability, improved drug bioavailability by 1.98 times. These results highlight the potential of bilosomes as an effective delivery system to enhance the stability and bioavailability of epigallocatechin gallate.	[178]
Luteolin	Thin film hydration	Cholesterol, span 60, bile salt, PEG 2000	Antioxidant, antimicrobial, and breast cancer	The selected PEGylated bilosomes have nanosize, higher encapsulation (~75.05), showed biphasic release, and greater permeation. Additionally, the nanocarriers showed higher antioxidant activity, greater cell viability on cancer cell lines, and superior antibacterial activity against Staphylococcus aureus and Escherichia coli compared to pure drug.	[179]
Lycopene	Lipid hydration	Span 60, cholesterol, sodium cholate	Antibacterial effect	The formulation showed strong antibacterial activity against multidrug-resistant *K. pneumoniae*. In vivo studies in a mouse lung infection model demonstrated significant therapeutic effects, including reduced lung inflammation and congestion, restored normal lung architecture, and decreased pulmonary fibrosis.	[180]
Psoralidin	Thin film hydration	Phosphatidylcholine, cholesterol, span 60, sodium deoxycholate	Breast and lung cancer	Developed formulation has shown better mucoadhesivity. In vitro studies revealed significantly improved apoptotic and necrotic effects on breast (MCF-7) and lung (A549) cancer cell lines, highlighting the potential of bilosomes as an effective oral treatment.	[181]
Sea-buckthorn pulp oil	Thin film hydration with ultrasonication	L-α-phosphatidylcholine, cholesterol, pluronic P123	Antioxidant, anti-inflammatory, wound healing, gastroprotective	Attenuated total reflection Fourier-transform infrared spectroscopy provided insights into bilosomes oil interactions. This study presents a reliable method to develop highly stable bilosome delivery systems with nanosize suitable for application in food and cosmetics.	[182]
Spirulina	Thin film hydration	Phosphatidylcholine, cholesterol, sodium deoxycholate	Photoaging effect	In vivo studies evaluated the photoprotective and antiaging effects of formulation through biochemical analysis of antioxidant, anti-inflammatory, and anti-wrinkling markers. Biochemical and histopathological findings confirmed that formulation offered superior antiaging benefits compared to pure drug.	[183]

**Table 6:** Phytochemical name, preparation technique, composition, and highlights of transferosomal, ethosomal, and transethosomal delivery of phytochemicals.

Phytochemical	Preparation Technique	Composition	Highlights	Disease/ Therapeutic Application	Ref.
Transferosomes					
Asiatic acid	High pressure homogenization	Sodium deoxycholate, tween 80	Small particle size (27.15–63.54 nm), high encapsulation (90.84%), and 71.65% anti-inflammatory activity were observed with the formulation. In clinical trials, the transfersomal gel showed no adverse effects, along with significant improvements in melanin index and skin elasticity at 2, 4, and 8 weeks, demonstrating its potential as an effective treatment for hypertrophic scars.	Hypertrophic scars	[191]
*Centella asiatica*	Thin film hydration	Soyaphosphatidyl choline, tween 80, propylene glycol	The *Centella asiatica* transfersomes and *Bergamot essential oil* nanoemulsions combination showed effectiveness in protecting against UVB-induced skin damage in BALB/c mice. It reduced oxidative stress by enhancing SOD activity, lowering MDA levels, and suppressing proinflammatory cytokines. It also promoted collagen production, highlighting its potential as a therapeutic agent for UVB-induced skin protection.	Antiphotoaging effects	[192]
*Curcuma comosa* extract	Thin film hydration	Phospholipon 90G, transcutol P, cremophor RH 40	Optimal transfersomes, containing diarylheptanoids dispersed in Carbopol gel followed zero order release kinetics and exhibited greater permeation. In vivo pharmacokinetics in rats showed a peak concentration of ~220 ng/mL and sustained plasma levels for over 12 h.	Estrogenic activity	[193]
Jabuticaba peel	Dispersion technique with ultrasonication	Lipoid S75, tween 20, hydroxyethyl cellulose, sodium hyaluronate	The extract was encapsulated in transfersomes, with or without polymer modifications (hydroxyethyl cellulose and sodium hyaluronate). Polymer-enriched transfersomes had larger sizes but were more stable and showed superior bioactivity compared to the extract solution, effectively reducing hydrogen peroxide toxicity and accelerating wound healing in cell models.	Antioxidant, wound healing	[190]
Mulberry leaf extract	Thin film hydration	Phospholipon 90G, tween 80	Optimized transferosome gel showed higher antioxidant activity, drug content, EE, ex vivo drug release, spreadability, homogeneity, and stability.	Antioxidant	[194]
Oleuropein and lentisk oil	Lipid hydration	Soy phospholipid, tween 80	Developed transferosomes formulation effectively reduced the overexpression of inflammatory markers, particularly MMP-1 and IL-6, mitigated oxidative stress induced by hydrogen peroxide, and accelerated wound healing in fibroblast monolayers in vitro.	Skin regeneration	[195]
Radish sprouts extract	Thin film hydration with sonication	Phospholipon 90G, polysorbate 20, 40, 60, and 80	The extract-loaded transferosomes reduce tyrosinase activity and melanin content while being safe for skin cells. Combined with sunscreen emulgels, the formulation enhanced cytotoxic safety, demonstrated stability, provided effective UVA and UVB protection and prolonged efficacy.	UV protection and antiaging effect	[196]
*Solanum xanthocarpum* methanolic extract	Thin film hydration	Phospholipon 90G, cholesterol, sodium cholate	The developed formulation showed nanosize, high entrapment, loading and antioxidant activity. Ex vivo studies confirmed significantly better skin permeation (82.86%) and retention compared to the conventional gel (35.28%), highlighting its potential for effective topical delivery.	Psoriasis	[197]
*Rhodomyrtus tomentosa* leaf extract	Thin film hydration	L-α-phosphatidylcholine, cholesterol, tween 80, tween 20, span 80, span 20, sodium deoxycholate	Spherical nanovesicles (405.3 ± 2.0 nm), high encapsulation efficiency (81.90 ± 0.31%), low polydispersity index (0.16 ± 0.08), and stable zeta potential (−61.62 ± 0.86 mV). The MIC and MBC values ranged from 8 to 256 and 64 to 1024 μg/mL, respectively. Exhibited strong antioxidant activity against DPPH and ABTS radicals and moderate tyrosinase inhibition. A significant reduction in nitric oxide production (6.78–88.25%) was observed.	Soft tissue infections	[188]
Syringic acid	Ethanol injection	Phosphatidylcholine, tween 80, d-α-tocopheryl polyethylene glycol 1000 succinate, diphenyl-1-picrylhydrazyl	Optimized transferosomes demonstrated significant antioxidant activity (IC_50_ = 11.1 µg/mL), and excellent skin deposition (78.72%). Additionally, it reduced acne lesions by 79.5%, outperforming Adapalene^®^ gel (18.7% reduction) without causing irritation or erythema, highlighting its potential as an effective and skin friendly acne treatment.	Acne	[198]
Ethosomes					
*Achillea millefolium* L. extract	Cold	Lipoid, propylene glycol, ethanol	Optimized ethosomal carrier demonstrated 88% free radical scavenging activity, along with high phenolic and flavonoid contents. The topical gel was stable and showed release of 79.8%.	Antioxidant	[199]
*Arctostaphylos uva-ursi* extract	Lipid dispersion	Phospholipid, ethanol, propylene glycol	Optimized formulations possess all pharmaceutical characteristics. The gel formulation demonstrated significant effects in reducing skin erythema, melanin, and sebum levels while enhancing skin hydration and elasticity.	Skin rejuvenation and depigmentation	[200]
Berberine chlride and evodiamine	Modified single step injection technique	Soybean lecithin, cholesterol, ethanol, propylene glycol	The developed formulation showed highest drug deposition in the epidermis cell viability tests confirmed that the optimized ethosomes enhanced the inhibitory effect on B16 melanoma cells.	Melanoma	[201]
*Brassicaceae* extract	Cold	Phospholipon 90 G, cholesterol, ethanol	Optimized ethosomal gel showed characteristics suitable for skin application. The release of bioactives follows the Korsmeyer–Peppas model and shows higher flux values.	Photoprotectiion	[202]
Caffeic acid	Lipid dispersion	Soybean lecithin, pluronic F127	Permeability of caffeic acid increased drastically when formulated as ethosomes. Skin-covered oxygen electrode experiments confirmed improved delivery and antioxidant activity of caffeic acid to porcine skin compared to a simple solution.	Antioxidant	[203]
*Phyllanthus nruri*, croton tiglium, zingiber officinale extract	Solvent dispersion with probe sonication	Soya lecithin, ethanol	Studies in cell line (HaCaT) revealed crude extracts-loaded ethosomal formulation inhibits testosterone and improves the cell viability similar to minoxidil. Preclinical safety was confirmed through in vitro cytotoxicity and histopathological studies.	Antiandrogenic and cytoprotectant	[204]
*Sambucus nigra* L. extract	Lipid hydration	Soybean phosphatidylcholine, ethanol	Prepared ethosomes showed slow release profile over 24 h and exhibited collagenase inhibition activity and excellent skin compatibility in human applications. These results indicate the potential of extract-loaded ethosomes as a promising cosmeceutical ingredient for skin care.	Skin care	[205]
*Vernonia anthelmintica* (L.) Willd.	Injection with ultrasonication	Soybean lecithin, cholesterol, ethanol	Ethosome formulation exhibited good physicochemical properties. In vitro studies showed significant therapeutic efficacy against vitiligo caused by hydroquinone exposure. Histological analysis of mouse skin showed increased melanin, elevated enzyme activities, and reduced antioxidant levels.	Vitiligo	[206]
Transethosomes					
Apigenin	Thin film hydration	Soya phospholipid, span 80	Optimized formulation demonstrated good EE, low vesicle size, and greater drug release. Ex vivo permeation showed superior apigenin delivery while cytotoxicity studies confirmed that formulation significantly reduced cell viability more effectively than the conventional gel.	Skin cancer	[207]
*Chenopodium murale* extract	Injection	Soybean phosphatidylcholine, cholesterol, tween 80	Drug-loaded transethosomes exhibited good physicochemical properties. The formulation maintained the extract’s macrostructural integrity, enabling enhanced skin permeation and achieving higher drug release.	Anti-inflammatory, antimicrobial, antioxidant and wound healing	[208]
Colchicine	Cold	Phospholipon 90G, tween 20, sodium taurocholate, or labrafil	Formulation was optimized by 2^2^ full factorial design and has exhibited desirable properties for transdermal drug delivery. Ex vivo studies confirmed higher flux compared to normal gel and demonstrated satisfactory stability.	Gout, and familial Mediterranean fever	[209]
Fisetin	Lipid film hydration with ultrasonication	Lipoid S100, sodium cholate	Transethosomes vesicles showed suitable physicochemical properties for transdermal delivery. In vivo data indicated that the formulation fluidized the rigid membranes of rat skin and deeper skin penetration.	Skin cancer	[210]
Ginger extract	Cold injection	Phospholipon 90G, cholesterol	Transethosomal formulation demonstrated enhanced flux and skin deposition compared to free drug hydrogel. In vivo studies confirmed significant edema reduction by the transethosomal hydrogel compared to free drug and ketoprofen gels. Treated animals exhibited marked decrease in reactive oxygen species and prostaglandin E2 levels.	Anti-inflammatory effect	[211]
Glycyrrhizic acid	Lipid film hydration	Phospholipid 90G, sodium cholate	Transethosome was optimized using the Box–Behnken design, which exhibited ideal characteristics for skin application. Ex vivo data demonstrated enhanced permeation and distribution of the drug, which highlighted superior skin deposition and retention of the formulation compared to conventional gels.	Skin cancer	[212]
Hexatriacontane	Lipid film hydration	Lipoid S 100, sodium cholate	Developed formulation showed optimized characteristics and Higuchi release kinetics. Dermatokinetic studies confirmed higher drug deposits in epidermal layers. Formulation exhibited effective antibacterial activity against *S. aureus* and *E. coli*.	Skin infections	[213]
Sinapic acid	Thin film hydration	Phospholipon 90 G, sodium deoxycholate	Optimized transethosome formulation demonstrated characteristics ideal for skin delivery. Higher antioxidant activity and greater penetration across the Strat-M^®^ membrane were observed.	Anti-inflammatory, antioxidant, anticancer, and antibacterial activities	[214]

**Table 7:** Phytochemical name, preparation technique, composition, and highlights of cubosomal delivery of phytochemicals.

Phytochemical	Preparation Technique	Composition	Disease/Therapeutic Application	Highlights	Ref.
β-carotene	High pressure homogenization	Glyceryl monooleate, poloxamer 407	Antioxidant	Design of experiment-based optimized formulation has 1800 mg glyceryl monooleate and 200 mg poloxamer 407. The optimized formulation demonstrated higher drug release. Incorporating β carotene into cubosomal formulations enhanced ~2-fold in its antioxidant potential.	[239]
*Brucea javanica* oil, doxorubicin hydrochloride	High pressure homogenization	Glycerol monooleate, pluronic F127	Antitumor	pH-responsive cubosomes exhibited phase transitions under different pH conditions and enhanced interaction in acidic tumor environments. Demonstrated antitumor activity against MCF-7 and doxorubicin-resistant MCF-7 cells, with the potential to overcome doxorubicin resistance.	[240]
Curcumin, fish oil	Thin film hydration	Monoolein, PEG 1000	Neuroprotection	Optimized cubosome showed negligible cytotoxicity at concentrations of 300 and 500 nM. In neuronally derived SH-SY5Y cells, this formulation effectively mitigated hydrogen peroxide-induced oxidative stress by reducing intracellular ROS levels and apoptosis. Higher neuroprotective potential noticed here suggests combination treatments in neurodegenerative disorders.	[241]
Erucin	Solvent evaporation	Monoolein, pluronic 84	Antioxidant and antiproliferative activities	The antioxidant potential of the developed vesicle was evaluated using DNA nicking, DPPH, and phosphomolybdate assays, and demonstrated significantly improved results compared to erucin alone. Additionally, it exhibited enhanced anticancer activity with a lower IC_50_ value in MTT assays.	[242]
Phycocyanin	Emulsification with homogenization	Glyceryl monooleate, poloxamer 407	Antioxidant	Optimized formulation is ideal for skin therapy, better stability and drug release followed the Higuchi model. Drug formulation showed superior skin permeability compared to the phycocyanin solution and was readily taken up by keratinocytes, providing prolonged antioxidative effects.	[243]
*Ruta graveolens* extract	Hot emulsification	Glyceryl monooleate, pluronic F108	Anti-asthmatic	Cubosomal formulation reduced asthma scores and improved lung function by restoring the FEV1/FVC ratio to normal levels. The nanocarrier demonstrated strong antioxidant and anti-inflammatory effects by reducing malondialdehyde, IL-4, IL-7, TGF-β, and Ig-E levels while increasing superoxide dismutase and INF-γ levels in bronchoalveolar lavage fluid.	[238]
Withanolide A	Hot emulsification	Glycerol monooleate, poloxamer 403, pluronic F127	Anti-diabetic, anti-neuropathic, anti-inflammatory, and anti-bacterial activities	Developed formulation showed superior antidiabetic, antineuropathic, anti-inflammatory, and antibacterial activities than the drug alone. Effects like HbA1c normalization, insulin secretagogue potential, oxidative stress reduction, and modulation of pro-inflammatory cytokines indicate the potential of this carrier in oral therapy.	[244]
*Yucca filamentosa*	Hot emulsification	Glyceryl monooleate, pluronic F108	Gastroprotective effect	The formulation showed superior gastroprotective, antioxidant, and anti-inflammatory effects in rat models of ethanol induced gastric injury and was comparable to famotidine. Developed formulations demonstrated potential in preventing peptic ulcer recurrence through modulation of the HMGB-1/RAGE/TLR4/NF-κB pathway.	[245]

**Table 8:** Overview of preparation techniques, excipients, and stability characteristics of different vesicular nanocarriers.

Vesicular Nanocarrier	Preparation Techniques	Commonly Used Excipients	Key Features and Stability	Ref.
Liposomes	Thin film hydration, electroformation, vesicle fusion, solvent injection, detergent dialysis, reverse phase evaporation, solvent spherule, size reduction, microfluidic, supercritical fluid, freeze-drying of double emulsions, membrane contactor method.	Phospholipids (e.g., phosphatidylcholine), cholesterol, cryoprotectants (e.g., trehalose)	Encapsulate hydrophilic and lipophilic drugs. Stability: Sensitive to oxidation, requires antioxidants or lyophilization for improved stability.	[246]
Invasomes	Thin film hydration, sonication, extrusion	Phospholipids, cholesterol, terpenes (e.g., cineole, limonene)	Enhanced dermal and transdermal drug delivery via terpene-mediated membrane fluidization. Stability: Moderate; terpene volatility may impact long-term stability.	[248]
Niosomes	Thin film hydration, ether injection, micro fluidization, reverse phase evaporation	Non-ionic surfactants (e.g., Span 60, Tween 80), cholesterol	Cost-effective alternative to liposomes. Stability: More stable than liposomes; however, sensitive to temperature and pH changes.	[249]
Bilosomes	Thin film hydration, sonication, extrusion	Bile salts (e.g., sodium deoxycholate), phospholipids, cholesterol	Improved oral bioavailability via bile salt stabilization. Stability: Enhanced resistance to gastrointestinal degradation; stable under physiological pH.	[165,250]
Transferosomes	Thin film hydration with edge activators, ultrasonic dispersion, ethanol injection	Phospholipids, edge activators (e.g., sodium cholate, span 80), ethanol	Highly deformable vesicles for transdermal delivery. Stability: Sensitive to environmental conditions like ethanol evaporation or oxidation.	[251]
Ethosomes	Hot or cold method, sonication, extrusion	Ethanol, phospholipids (e.g., phosphatidylcholine), propylene glycol	Enhanced skin penetration. Stability: Sensitive to ethanol evaporation; proper sealing is needed to maintain integrity.	[252]
Transethosomes	Combination of ethosomes and transferosomes techniques, sonication, extrusion	Phospholipids, ethanol, edge activators (e.g., Tween 80)	Combines properties of ethosomes and transferosomes. Stability: Sensitive to ethanol loss and oxidation; requires proper storage.	[221,253]
Cubosomes	Top-down approach, bottom-up approach	Lipids (e.g., glyceryl monooleate), polymers (e.g., pluronic F127)	High internal surface area for drug encapsulation. Stability: More stable than vesicular systems; sensitive to dilution and surfactant imbalance.	[229]

**Table 9:** Typical in vitro and in vivo tests of vesicular carriers utilized for phytochemical delivery applications.

Test Type	Principle	Equipment Used	Evaluation Parameters	Ref.
Particle size analysis	Determines vesicle size distribution	Dynamic light scattering (DLS)	Particle size, polydispersity index	[271]
Zeta potential	Measures surface charge of vesicles	Zeta potential analyzer	Surface charge, colloidal stability	[272]
In vitro drug release	Measures drug diffusion through a membrane	Franz diffusion cell, dialysis membrane	Drug release rate, cumulative release profile	[273]
Permeability	Simulates passive drug permeation	Permeability apparatus, membrane models	Permeability coefficient, flux rate	[274]
Stability testing	Assesses long-term stability	Stability chambers, DLS, HPLC	Particle size, drug retention, degradation products	[275]
Thermal stability	Evaluates stability under varying temperature conditions	Thermal analyzer, stability chamber	Degradation rate, active ingredient stability	[276]
Oxidative stability	Assesses stability in the presence of oxygen or reactive species	Oxidative chamber, spectrophotometer	Oxidation products, active ingredient retention	[277]
Photostability test	Assesses stability under ultraviolet (UV) or light exposure	UV chamber, spectrophotometer	Degradation products, remaining active phytochemical	[278]
Antioxidant assay	Evaluates antioxidant activity (e.g., DPPH, ABTS, FRAP, ORAC, FIC, CUPRAC)	UV–vis spectrophotometer	Percentage inhibition, antioxidant capacity	[279]
Anti-inflammatory assay	Measures inhibition of inflammatory mediators	Enzyme-linked immunosorbent assay (ELISA), cell culture models	Cytokine levels (e.g., TNF-α, IL-6)	[280]
Pharmacokinetic study	Measures drug absorption, distribution, metabolism, and excretion	HPLC, mass spectrometry	C_max_, T_max_, AUC, half-life	[281]
Biodistribution study	Analyzes drug accumulation in tissues	Fluorescent imaging, gamma counters	Organ-specific drug concentration, clearance	[282]
Therapeutic efficacy	Evaluate pharmacological effects (e.g., anticancer, neuroprotection)	Animal models, histopathology	Disease regression, biomarker levels	[283]
Cell viability	Assesses cell metabolic activity post-exposure (e.g., MTT)	Microplate reader, cultured cells	Cell viability, IC_50_	[284]
Oxidative stress markers	Analyzes ROS, lipid peroxidation, or protein oxidation	ELISA, biochemical assays	ROS levels, malondialdehyde, protein oxidation	[285]
Cytokine profiling	Measures inflammatory and immunomodulatory effects	ELISA, multiplex assays	Cytokine expression (e.g., IL-10, IFN-γ)	[286]
Antiviral activity assay	Evaluates inhibition of viral replication	Cell culture models, PCR	Viral load, cytopathic effect	[287]
Antimicrobial assay	Determines antimicrobial efficacy	Microdilution plates, colony counter	Minimum inhibitory concentration, zone of inhibition	[288]
Cardioprotective assay	Assesses protection against cardiac injury or dysfunction	Animal models, echocardiography	Cardiac biomarkers, ECG, histology	[289]
Neuroprotective assay	Evaluate protective effects against neuronal damage	Neuronal cell lines, animal models	Neuronal viability, ROS levels, neuroinflammation markers	[290]
Anticancer assay	Measures inhibition of cancer cell growth	MTT assay, flow cytometry	Cell viability, apoptosis rate, tumor volume	[291]
Hepatoprotective assay	Assesses protection against liver damage	Animal models, liver function tests	Liver enzymes (ALT, AST), histopathology	[292]
Immunomodulatory assay	Evaluates modulation of immune response	ELISA, multiplex cytokine assays	Cytokine profile, immune cell activation	[293]
Antidiabetic assay	Measures blood glucose regulation and insulin sensitivity	Animal models, glucometer	Blood glucose, insulin levels, HbA1c	[294]

**Table 10:** A compilation of recently filed patents for pharmaceutical nanosuspensions, highlights of their innovation.

Application ID	Publication Date	Title	Summary of Invention	Potential Benefits
11868251	5 October 2007	Liposomal curcumin for treatment of neurofibromatosis	The invention presents a method to treat neurofibromatosis types 1 and 2 using curcumin or its analogues encapsulated in colloidal drug delivery systems, such as liposomes, nanoparticles, or micelles. The system targets Merlin and related pathway proteins and is administered parenterally with a pharmaceutically acceptable carrier.	Neurofibromatosis
WO/2009/061787	14 May 2009	Coated devices and method of making coated devices that reduce smooth muscle cell proliferation and platelet activity	The invention features drug eluting stents and coated devices that release resveratrol and quercetin to maintain blood flow, reduce smooth muscle cell proliferation, and limit restenosis.	Atherosclerosis, stenosis, and clotting disorders.
201010158483.X	22 March 2010	Allicin liposome for resisting microbe, protozoan and tumor and preparation method thereof	The invention introduces an allicin liposome formulation to treat microbes, protozoa, tumors, and viruses. Its enhanced properties expand allicin’s applications beyond bacterial and fungal infections, offering new therapeutic possibilities.	Antimicrobial, protozoal and viral infection
201210041878.0	23 February 2012	Wogonin liposome preparation modified with glycyrrhetinic acid and preparation method thereof	The invention presents a glycyrrhetinic acid-modified wogonin liposome, prepared through the reverse evaporation method, which enhances efficacy against liver tumor cells and improves liver cancer treatment.	Hepatic carcinoma
14777160	15 March 2013	Compositions and methods including celecoxib and plumbagin relating to treatment of cancer	The invention presents a method for treating proliferative diseases, especially skin cancer, using a combination of celecoxib and plumbagin for improved therapeutic outcomes.	Skin cancer
WO/2016/005786	14 January 2016	Liposomal formulations comprising thymoquinone and taxane, and methods of treating cancer using same	The invention describes liposomal compositions with taxane and thymoquinone, offering synergistic anticancer effects, improved drug encapsulation, enhanced stability, and consistent taxane release for more effective cancer treatment.	Synergistic Anticancer effects
201711105675.2	10 November 2017	Brain-targeting liposome with paeonol-ozagrel conjugate and preparation method thereof	The invention describes a brain-targeting liposome containing a paeonol-ozagrel conjugate, designed to enhance drug delivery across the BBB. It prolongs drug circulation time and enables targeted treatment of various thrombotic cardiovascular and cerebrovascular diseases.	cardiac and cerebral ischemia or infarction
202311320134.7	12 October 2023	Echinacoside liposome, LRP1 targeted echinacoside-loaded liposome, and preparation method and application of echinacoside-loaded liposome and LRP1 targeted echinacoside-loaded liposome	The invention features an LRP1-targeted echinacoside liposome that enhances brain delivery, improves dopamine levels, reduces oxidative stress, and restores motor function in neurodegenerative disease models.	Neurodegenerative diseases
202311327683.7	13 October 2023	Aptamer-macrophage membrane composite nano delivery system for targeting breast cancer and method for analyzing anti-breast cancer action mechanism	The invention describes a nano-delivery system utilizing betulinic acid encapsulated in macrophage membranes modified with nucleic acid aptamers for targeted breast cancer treatment. Betulinic acid effectively inhibits cancer cell proliferation and induces apoptosis by blocking the PI3K/AKT and MAPK signaling pathways.	Breast cancer

## Data Availability

The data presented in this study is contained within this article.
